# Peak pressure experienced by the human hand during suspension and vertical climbing

**DOI:** 10.1242/bio.062562

**Published:** 2026-08-20

**Authors:** Victoria A. Lockwood, Szu-Ching Lu, Samantha L. Winter, Hui Yang, Tracy L. Kivell

**Affiliations:** ^1^ Department of Anthropology, Center for the Advanced Study of Human Paleobiology, The George Washington University, Washington, DC 20052, USA; ^2^ PALEVOPRIM: Laboratoire de Paléontologie, Evolution, Paléoécosystèmes et Paléoprimatologie, Université de Poitiers, CNRS, Poitiers, 86073, France; ^3^ The Strathclyde Institute of Education, Faculty of Humanities and Social Sciences, University of Strathclyde, Glasgow, G4 0LT, UK; ^4^ National Center for Sport and Exercise Medicine, School of Sport, Exercise and Health Sciences, Loughborough University, Loughborough, LE11 3TU, UK; ^5^ School of Sport and Exercise Sciences, University of Kent, Medway Building, Chatham Maritime, Chatham, Kent, ME4 4AG, UK; ^6^ Institute for Research in Astrophysics and Planetology (IRAP), CNRS, Toulouse 31400, France; ^7^ Department of Physics, The George Washington University, Washington, DC 20052, USA; ^8^ Department of Human Origins, Max Planck Institute for Evolutionary Anthropology, Leipzig, 04103, Germany

**Keywords:** Peak pressure, Hand, Arboreal, Suspension, Vertical climbing, Support size

## Abstract

Reconstructions of potential arboreal capabilities in extinct human ancestors (hominins) are typically inferred from upper limb morphology. Arboreal environments are complex with multiple support sizes and orientations. However, data are limited on how arboreal support variables may affect hand loading during arboreal activities. Using a custom-built climbing frame with interchangeable support diameters (45 mm, 80 mm, 105 mm), we investigated the effects of support diameter and horizontal/vertical orientation on the pressure experienced by the human hand during postural and locomotor suspension and vertical climbing (*n*=14 participants each). Support diameter had a consistent effect on suspension and vertical climbing normalized peak pressure values, but thumb positioning or postural versus locomotor activities had no significant relationship. The 45 mm diameter had significantly greater normalized peak pressure values compared to the 80 mm and 105 mm diameters. Postural and locomotor activity differences were only observed during vertical climbing but varied with support diameter size. Suspension and vertical climbing frequently load the third ray but distal-proximal peak pressure location patterns differ with support orientation. Support diameter and, less so, orientation influence the magnitude of loading in the human hand during the investigated arboreal behaviors. Peak pressure location patterns can distinguish suspension and vertical climbing locomotor categories within our experimental setting.

## Teaser

**Summary:** Peak pressure values and detailed location patterns are identified in human hands during suspensory and vertical climbing activities in a simulated arboreal environment, with implications for hominin hand use.

## INTRODUCTION

Arboreality not only has an important role in the daily lives of extant non-human primates but also is hypothesized to have played a critical role in hominin evolution (e.g. Drummond-Clarke et al., 2022; Fleagle, 1976; Thorpe et al., 2007). For primates, including non-human apes, the arboreal environment provides, among other things, the primary food source (e.g. Chapman et al., 2003; Havmøller et al., 2021; Myatt and Thorpe, 2011; Wrangham, 1977), shelter (e.g. Fruth et al., 2018; Pozzi et al., 2022; Samson, 2012), and protection from predators (e.g. Seyfarth et al., 1980; but see Isbell, 1994). However, the arboreal environment is complex with multiple support sizes and orientations for primates to grasp during a variety of locomotor and postural behaviors (e.g. Manduell et al., 2012; McGraw, 1996). Hand grasping postures during arboreal locomotion have been studied in a few primates (for review, see Schmitt et al., 2016), including in African apes (Hunt, 1991; Neufuss et al., 2017; Samuel et al., 2018), which have, in turn, helped to inform functional inferences from hand morphology, both in extant and extinct taxa (e.g. Almécija et al., 2015; Susman, 1979; Syeda et al., 2023; reviewed in Kivell et al., 2023). However, the link between arboreal locomotion, grasping posture and hand morphology is hindered by the limited information on loads experienced by the hand during arboreal locomotion, including how these loads may vary with substrate size, orientation or locomotor behavior (Samuel et al., 2018). While several experimental studies have quantified human hand loading during object manipulation (e.g. Kong and Lowe, 2005; Seo et al., 2007; Vigouroux et al., 2011), including stone tool use (Williams-Hatala et al., 2018), or during rock climbing (Amca et al., 2012; Quaine et al., 2003), no study to date has explored hand loading during suspensory or non-rock climbing activities. Given the potential importance of arboreal locomotion in hominin evolution (Drummond-Clarke et al., 2022; Hunt, 1996; Thorpe et al., 2007), including in Pleistocene hominins (e.g. Georgiou et al., 2020; Kivell et al., 2015), we offer the first study, to our knowledge, to quantify hand loads and their specific location within the hand during simulated arboreal postures and locomotion (suspension and vertical climbing) to ultimately improve our understanding of hominin hand evolution and clarify the potential importance of arboreality in human evolution.

Hand morphology, either of extant or extinct taxa, is often used to provide insights into arboreal capabilities during hominid (i.e. great apes, including humans) (e.g. Almécija et al., 2015) and, particularly, hominin evolution (e.g. Kivell et al., 2023). Robust reconstruction of the potential functional signal within hand morphology requires knowledge of how the hand is loaded during specific activities. However, data on hand loading during arboreal behaviors in hominids is particularly challenging to collect, with only two studies, to our knowledge, to date: ‘simulated arboreal’ knuckle-walking on a 102 mm-diameter pole in captive chimpanzees (*Pan troglodytes*; Wunderlich and Jungers, 2009) and arboreal suspension, vertical locomotion and knuckle-walking on a 120 mm-diameter pole in zoo-housed bonobos (*Pan paniscus*; Samuel et al., 2018). In both studies, the third digit most often experienced peak pressure during all locomotor activities (Samuel et al., 2018; Wunderlich and Jungers, 2009). In bonobos during both suspension and vertical climbing, peak pressure was located in the distal digits and the proximal palm (Samuel et al., 2018). Whilst these similarities between locomotor categories suggest the potential for a consistent pattern in hand loading across extant hominids, how hand loading may vary in other hominid taxa and on smaller or larger diameter supports remains unknown. Given that great ape arboreal locomotion is highly varied in support orientation and size (Doran, 1993a,b; Doran and Hunt, 1994; Hunt, 1992; Hunt et al., 1996; Manduell et al., 2011; Remis, 1995), it is necessary to consider the effects of these variables to more fully infer the potential arboreal locomotor regimes of extinct hominins.

Locomotion involves displacement of body mass in the environment to a greater degree than posture (Prost, 1965), and multiple limbs may contact different supports at different points during the locomotor sequence (Manduell et al., 2011; Preuschoft and Demes, 1984; Thorpe et al., 2007). Thus, the contact area between the limbs and the support will be more variable during locomotion than during posture, leading to differences in the pressure experienced by each limb. Adjusting the contact area acted on by a force, or the magnitude of the vertical force applied to an object can alter the pressure experienced. Substrate reaction force (SRF) increases with increasing locomotor speed, leading to a greater force magnitude experienced by the limb in contact with the substrate (Alexander, 1984; Biewener, 1990; Patel and Wunderlich, 2010). This can lead to increases in pressure, which can be mitigated by increasing the contact area between the substrate and the limb (Patel and Wunderlich, 2010). Yet during the gait cycle the limb-substrate contact area changes. For example, during arboreal climbing and suspension, bonobos show a rolling touch-down pattern, such that they touch-down with digit V and the medial palm and progress to digit II and the lateral palm during the gait cycle (Samuel et al., 2018). The modern human hand contact pattern during arboreal locomotion is unknown. Hand grips during locomotion may change across the gait cycle, in comparison to static grips, leading to differences in hand-support contact area and pressure.

Increasing grip force and pressure leads to increased friction, which is considered more beneficial for vertical locomotion (e.g. climbing) compared with horizontal (e.g. suspension) in primates given the need to overcome the tangential gravity component on the former (Cartmill, 1974; Preuschoft, 2002). Within a diameter graspable by a single hand, decreasing the central angle between the contact points of the hand results in an increased tangential component and greater friction required for a stable grip (Cartmill, 1974). In addition to anatomical components, such as volar pads (Cartmill, 1974), the relative digit positioning may aid friction generation. Thumb position (adducted or abducted) may be used to reinforce a grip and adjust the pressure experienced by the hand whilst grasping an arboreal support but is influenced by support orientation and size. In non-locomotor captive conditions strepsirrhine manual (and pedal) grip forces were greatest on supports that allow optimal digit positioning but wild strepsirrhines often use smaller than optimal diameter supports (Granatosky et al., 2025). However, whole-body mechanics experienced during arboreal locomotion were not accounted for (Granatosky et al., 2025). Thus, additional data investigating hand loadings during arboreal locomotion are needed.

Despite the lack of arboreal locomotion data, human hand loading has been investigated in multiple other contexts (e.g. Amca et al., 2012; Seo et al., 2007; Williams-Hatala et al., 2018). In static, non-arboreal gripping activities in humans, the position of the digits relative to the rest of the hand affects the amount of grip force generated (Seo et al., 2007). Diameters close to the optimal diameter for a cylindrical object gripped by the human hand (30-40 mm: Ayoub and Presti, 1971; Edgren et al., 2004; Hall, 1997; Kong and Lowe, 2005; Seo et al., 2007; Seo and Armstrong, 2008) allow the fingertips to be positioned opposing the palm, resulting in the generation of greater grip forces than on larger diameters (Seo et al., 2007). Whilst the interaction between grasped object size and the hand has been extensively investigated in the ergonomic literature (e.g. Ayoub and Presti, 1971; Edgren et al., 2004; Hall, 1997; Kong and Lowe, 2005; Seo et al., 2007; Seo and Armstrong, 2008), it is yet to be specifically examined in an arboreal locomotion context.

Human rock-climbing studies currently provide the closest behavioral comparison for investigating how force is experienced by the human hand during arboreality (e.g. Amca et al., 2012; Fuss et al., 2013; Fuss and Niegl, 2006; Jensen et al., 2005; Morenas Martín et al., 2013). However, hand positions used during rock climbing predominantly contact the supports via the distal aspect of the digits (Watts, 2004), in contrast to great ape arboreal hand postures (e.g. Neufuss et al., 2017; Samuel et al., 2018). Within the commonly used rock-climbing postures, the second and third digits generate the greatest forces during grips, with the third digit generally generating the greatest force (Fuss and Niegl, 2006; Quaine et al., 2003).

Here we investigate for the first time peak pressure experienced by the human hand during arboreal-like posture and locomotor activities on both vertical and horizontal supports of differing diameters. Peak pressure was selected to allow the greatest comparison to previous studies of hand pressure during evolutionary relevant behaviors, such as tool manufacture/use and locomotion (e.g. Samuel et al., 2018; Williams et al., 2012; Patel and Wunderlich, 2010; Wunderlich and Jungers, 2009; Williams-Hatala et al., 2018). We asked 14 participants for each set up (26 participants in total) to engage in climbing and/or suspensory posture and locomotion on a custom-built climbing frame with instrumented (pressure) sleeves of differing diameters whilst using kinematic methods to spatially locate their hands. Based on the previous literature in humans and great apes, we test the following hypotheses:

1. Normalized (i.e. standardized by participant hand area and body mass) peak pressure will vary by activity category (i.e. climbing versus suspension/locomotion versus posture) and diameter. Predication 1a): Dynamic locomotor activities will have a greater normalized peak pressure than the static postural activities for both suspension and vertical climbing. Prediction 1b): In both suspension and vertical climbing posture and locomotion normalized peak pressure will decrease as the diameter increases.
2. Location of peak pressure will be similar for both suspension and vertical climbing activities. Prediction 2a): Peak pressure location will be located on the third ray. Prediction 2b): Peak pressure location will be observed in the distal digits and proximal palm regions of the hand.
3. Thumb position will influence normalized peak pressure. Prediction 3a): An abducted thumb will have greater normalized peak pressure values compared to an adducted thumb for both suspension and vertical climbing posture and locomotion.

## RESULTS

Table 1 summarizes participant biometric data for suspension and vertical climbing, respectively (full data in Table S1).

**Table 1.** Participant body mass and hand size for suspension (*n*=14) and vertical climbing (*n*=14) activities. ‘s.d.’, standard deviation

Sex	Mean body mass (kg) [s.d.]	Body mass (kg) range	Mean hand length (mm) [s.d.]	Hand length (mm) range
Suspension				
Female (*n*=6)	61.7 [±5.3]	55.5-69.0	175.2 [±5.3]	169.8-184.2
Male (*n*=8)	82.6 [±11.1]	69.0-97.5	193.1 [±5.0]	187.7-200.9
Vertical climbing				
Female (*n*=8)	63.7 [±14.8]	47.5-88.0	171.6 [±8.2]	163.6-184.2
Male (*n*=6)	85.8 [±12.5]	69.0-102.0	188.7 [±4.9]	181.2-196.5

### Whole hand on or over the edge of the pressure mat

For suspension (433 trials, *n*=14) no significant difference (U=9029.5, *P*=0.115) was found in normalized peak pressure between trials with the whole hand on the mat and those with part of the hand over the edge of the pressure mat. For vertical climbing (609 trials, *n*=14) a significant difference (U=17,928, *P*=0.039) was found in normalized peak pressure for trials with the whole hand on the mat compared to those with part of the hand over the edge of the pressure mat. Thus, subsequent vertical climbing analyses only used trials where the whole hand was on the pressure mat (530 trials).

### Normalized peak pressure

Normalized peak pressure data for suspension and vertical climbing are presented in Table 2 (see Table S2 for raw peak pressure data). The descriptive statistics are calculated from participant activity-diameter combination mean values for suspension (121 trials, *n*=14) and vertical climbing (125 trials, *n*=14).

**Table 2.** Normalized peak pressure mean, standard deviation (s.d.), and range values for suspension (121 trials, *n*=14) and vertical climbing (125 trials, *n*=14)

Diameter (mm)	Activity	Mean normalized peak pressure (kPa/mm^2^/kg) [s.d.]	Range
Suspension			
45	Thumb adducted	1.5 [±0.5]	0.8–2.3
	Thumb abducted	1.7 [±0.5]	0.7–2.6
	Dynamic	1.4 [±0.5]	0.7–2.3
80	Thumb adducted	1.0 [±0.3]	0.7–1.7
	Thumb abducted	1.0 [±0.3]	0.5–1.7
	Dynamic	1.0 [±0.3]	0.5–1.6
105	Thumb adducted	1.0 [±0.3]	0.6–1.7
	Thumb abducted	0.9 [±0.3]	0.5–1.5
	Dynamic	0.9 [±0.3]	0.4–1.4
Vertical climbing			
45	Thumb adducted	0.5 [±0.2]	0.3–1.0
	Thumb abducted	0.6 [±0.3]	0.3–1.1
	Dynamic	0.7 [±0.3]	0.3–1.5
80	Thumb adducted	0.4 [±0.2]	0.1–0.9
	Thumb abducted	0.5 [±0.2]	0.2–0.9
	Dynamic	0.5 [±0.3]	0.1–1.1
105	Thumb adducted	0.4 [±0.2]	0.2–0.8
	Thumb abducted	0.5 [±0.2]	0.2–0.8
	Dynamic	0.5 [±0.2]	0.3–0.9

Normalization of peak pressure values was performed by dividing by the calculated value of hand area (third ray x palm width, mm^2^) divided by body mass (kg).

#### Suspension

On all three diameters, there was no difference in normalized peak pressure between the static thumb adducted and abducted activities and thus a mean static value for each participant per diameter was calculated. On each of the three diameters there were no differences in normalized peak pressure between the mean static value and the dynamic activity value. Within each diameter there were no differences in normalized peak pressure between the separate (static thumb adducted, static thumb abducted, and dynamic) activities.

A combined activity normalized peak pressure value per diameter was then calculated and a significant difference between diameters was found [X^2^(2)=16.91, *P*=<0.001]. Between diameter comparisons were conducted via a Nemenyi post-hoc test with single step *P*-value adjustment. Significant differences in combined activity normalized peak pressure were found between the 45 mm and 105 mm (*P*=<0.001) and 45 mm and 80 mm (*P*=0.004) diameters, with the 45 mm diameter having the greatest median value. No differences were found for the 80 mm and 105 mm diameter pairing.

Significant differences were also found between diameters when the activities were considered separately [static thumb adducted: X^2^(2)=18.43, *P*=<0.001, static thumb abducted: X^2^(2)=20.46, *P*=<0.001, and dynamic: X^2^(2)=12.17, *P*=0.002]. Comparisons between diameters were undertaken via Nemenyi post-hoc test with single step *P*-value adjustment and significant differences were found between the 45 mm and 105 mm diameters for each activity (static thumb adducted: *P*=0.001, static thumb abducted: *P*=<0.001, and dynamic: *P*=0.002). Significant differences between the 45 mm and 80 mm diameters were also found in the static thumb adducted (*P*=<0.001) and static thumb abducted (*P*=0.003) activity categories respectively. The greatest median value was on the 45 mm diameter in all cases. The remaining diameter pair combinations had no differences.

#### Vertical climbing

The static thumb adducted and abducted activity categories had significant differences in normalized peak pressure on the 45 mm diameter (Z=−2.47, *P*=0.040), with the abducted thumb condition having the greater mean value. The 80 mm and 105 mm had no differences in normalized peak pressure values between the static thumb adducted and abducted activity categories. Thus, mean static values were calculated for the 80 mm and 105 mm diameters and compared to the respective dynamic values. No difference was found between mean static and dynamic normalized peak pressure values for either diameter.

Within diameter comparisons of normalized peak pressure across the three activity categories, the 45 mm [X^2^(2)=7.00, *P*=0.045] and 80 mm [X^2^(2)=7.89, *P*=0.045] diameters had significant differences. No difference was found on the 105 mm diameter. Comparisons between activity pairs via Nemenyi post-hoc tests with single step *P*-value adjustments found significant differences in normalized peak pressure values between the static thumb adducted and dynamic activities on both the 45 mm (*P*=0.037) and 80 mm (*P*=0.037) diameters. The dynamic activity had the highest median value for both diameters. On the 80 mm diameter the static thumb adducted activity was significantly different from the static thumb abducted activity (*P*=0.048), with the static thumb abducted condition having the greater median value. The static thumb abducted and dynamic activities had no significant differences in normalized peak pressure values for vertical climbing.

Comparisons within activity categories and across diameters found significant differences in normalized peak pressure for each of the three activity categories [static thumb adducted: X^2^(2)=8.71, *P*=0.013, static thumb abducted: X^2^(2)=11.69, *P*=0.009, dynamic vertical climbing: X^2^(2)=8.71, *P*=0.013]. Post-hoc comparisons using Nemenyi post-hoc tests with single step *P*-value adjustment found significant differences in normalized peak pressure between the 45 mm and 105 mm diameters for the static thumb adducted (*P*=0.037), static thumb abducted (*P*=0.005), and dynamic (*P*=0.022) activity categories. Between the 45 mm and 80 mm diameters, significant differences in normalized peak pressure were also found for all three activity categories (static thumb adducted: *P*=0.022, static thumb abducted: *P*=0.017, and dynamic: *P*=0.037). The 45 mm diameter had the greatest median normalized peak pressure value for each comparison. For all three activity categories there were no differences between the 80 mm and 105 mm diameters.

### Coefficient of variation by activity and diameter

Coefficients of variation were calculated using the mean for each activity-diameter combination. For both suspension and vertical climbing, no difference in the variance was found among the three activities within each diameter or among the three diameters within each activity (see Table S3).

### Location of peak pressure

**Fig. 1.**
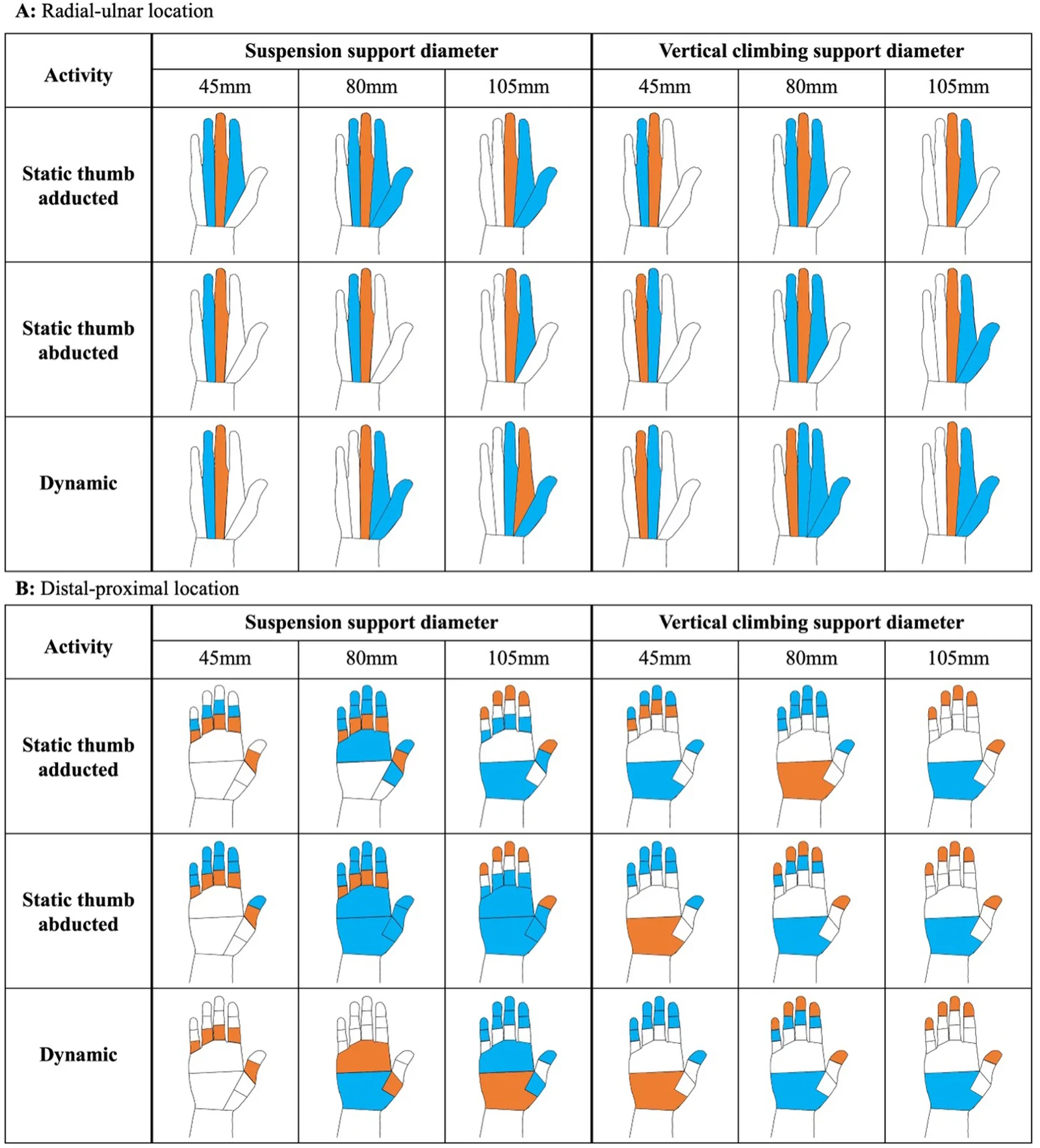
**Locations of peak pressure within the hand during suspension and vertical climbing activities on 45 mm, 80 mm, and 105 mm diameter supports.** (A) Within hand radial-ulnar peak pressure location for all activity-diameter combinations in suspension (*n*=402 trials) and vertical climbing (*n*=436 trials). (B) Within hand distal-proximal peak pressure location for all activity-diameter combinations in suspension (*n*=373 trials) and vertical climbing (*n*=457 trials). Blue: location in 10.0% or more of all usable trials for that activity-diameter combination. Orange: mode location for all usable trials for each activity-diameter location.

For all usable trials the percentage of each radial-ulnar and proximal-distal peak pressure location were calculated for each activity-diameter combination for suspension and vertical climbing, respectively (see Tables S4-S7). To account for sample noise only locations that occurred at a frequency of 10.0% or above were included. The radial-ulnar results are shown in Fig. 1A, and the proximal-distal results are shown in Fig. 1B. Locations occurring at a frequency of 10.0% or higher are coded in blue and the mode (most frequent) location for each activity-diameter combination is in orange.

The radial-ulnar most frequent mode location was the third ray, as it was the mode location for all but one suspension and three vertical climbing activity-diameter combinations. Thus, the trend is most consistent within suspension. After the third ray the second ray was the next highest frequency radial-ulnar location for suspension, whereas within vertical climbing it was the fourth ray (see Fig. 1A).

Within the distal-proximal analysis, the mode location for suspension moves from the proximal phalanges to the distal phalanges and metacarpal bases as the diameter increases (see Fig. 1B). In contrast, for all vertical climbing activity-diameter combinations the mode location is at the proximal-most or distal-most parts of the hand and moves to just the distal phalanges on the largest diameter (see Fig. 1B).

The trends observed in the modal analysis above were also generally, yet weakly, supported in the multinomial logistic regression risk ratios (see Tables S8-S11), as the adjusted R^2^ values were small (suspension radial-ulnar: 0.208, vertical climbing radial-ulnar: 0.219, suspension distal-proximal: 0.265, vertical climbing distal-proximal: 0.128). This indicates that additional variables beyond diameter and activity impact the location of peak pressure within the human hand during suspension and vertical climbing.

## DISCUSSION

### Dynamic versus static activities

Our prediction that dynamic activities would have greater median normalized peak pressure values (hereafter, ‘peak pressure’) compared to static activities was partially supported. The observed dynamic-static differences are likely the result of differences in hand-support contact area between postural (more consistent contact area) and locomotor (changing contact area) activities. However, hand contact area has been shown to be modifiable during terrestrial quadrupedal locomotion at various speeds (Patel and Wunderlich, 2010). At higher travel speeds, and thus greater ground reaction force magnitudes, the terrestrially digitigrade olive baboon (*Papio anubis*) used palmigrade hand postures resulting in lower-than-expected pressure values (Patel and Wunderlich, 2010). In the current study the lack of differences between static and dynamic activities on the largest diameter suggests potential differences in hand posture for dynamic and static activities between these diameter groups.

### Substrate diameter effect

Our prediction that peak pressure will decrease as with increasing diameter size, and that this is applicable across multiple support orientations, was generally supported with vertically oriented support size having the greatest effect on the dynamic activity category.

Our finding of the highest peak pressure on the 45 mm diameter during both suspension and vertical climbing is in accordance with ergonomic literature showing the generation of greater grip forces on cylindrical objects with an optimal diameter for grasping by the human hand (30-40 mm) relative to larger-diameter objects (Hall, 1997; Kong and Lowe, 2005; Seo et al., 2007; Seo and Armstrong, 2008). This indicates the potential for applying an ergonomic approach to the study of arboreal support use in humans and potentially non-human primates (adjusted for relative hand size and proportions). The increased peak pressure on the 45 mm diameter is likely the result of the ability to position the distal phalanges opposite the distal aspect of the palm during the grasp, with the distal phalangeal force leading to increased palmar reaction forces and ultimately greater overall grip force (Seo et al., 2007). With increasing support diameter, the digits are likely extended with the distal phalanges being positioned further away from and no longer in opposition to the proximal palm. See Fig. S1 for illustration. In addition to finger flexion when grasping an object, the position of the wrist is an important consideration in generating grip force and subsequently pressure. In human non-locomotor contexts, there is a synergistic relationship between the flexion and extension of the wrist and the manual digits (Su et al., 2005). Gibbons have also been shown to increase wrist flexion when grasping larger suspensory supports (Sarmiento, 1988). Whilst how increases in support size impact wrist positioning during arboreal locomotion has yet to be investigated in humans, it suggests that arboreal support size likely impacts hand positioning and thus the grip force (and subsequently pressure) generating ability of the human hand. Further kinematic study of the joint angles of the human hand within the context of the upper limb during arboreal activities on varying support sizes is needed to fully investigate the extent of this ‘kinematic chain’.

### Peak pressure location

Our prediction that peak pressure location would be found on the third ray was generally supported. Across all diameters for both suspension and vertical climbing, the third ray was most often the radial-ulnar mode location of peak pressure, followed by the second ray for suspension and the fourth ray for vertical climbing (see Fig. 1A). However, only partial support was found for our predication that peak pressure would occur at the proximal palm and distal phalanges. During suspension the distal-proximal peak pressure location mode moved from the proximal phalanges to the distal phalanges and proximal palm with increasing diameter, but for vertical climbing it remained at the distal phalanges or proximal palm regardless of diameter (Fig. 1B). This suggests the potential for using within ray morphological variation for investigating specific arboreal capabilities within primates and extinct hominins; for example, bony evidence of high loading on the proximal phalanges could indicate suspension on small diameters. However, the small adjusted R^2^ values suggest that additional factors may further explain the variation in the multinomial logistic regression models. Whilst this issue should be addressed with additional methods in future studies, general patterns for both suspension and vertical climbing were observable in our data.

### Thumb position

Our prediction that an abducted thumb position would result in increased peak pressure relative to the adducted thumb position was only partially supported, specifically during vertical climbing. An abducted (and opposed) thumb can dorsally reinforce the digits when gripping a static, non-arboreal, cylindrical object as it increases in length and mass (Napier, 1956). Whilst the size of the diameters used in our study did not permit dorsal reinforcement of the digits by the thumb, the abducted thumb position may have permitted some grip reinforcement.

### Human and bonobo comparison

The largest diameter (105 mm) support in our study had a hand length to support circumference ratio that was comparable to that of the zoo-housed bonobo experimental study by Samuel et al. (2018). Thus, suspension and vertical climbing hand peak pressure locations could be compared for humans and bonobos.

During bonobo suspension and vertical climbing, peak pressure was most frequently located in the proximal palm and distal phalanges, particularly around the third ray, while little to no pressure was recorded for the thumb (Samuel et al., 2018). This pattern is similar to the distal-proximal mode peak pressure locations found in human suspension and vertical climbing on the 105 cm diameter in our study (see Fig. 1B). When all loading is considered, in suspension both humans (this study) and bonobos (Samuel et al., 2018) had minimal loading on the intermediate phalanges in comparison to the distal and proximal phalanges and the palm. Moreover, during vertical climbing both humans and bonobos showed minimal loading of the intermediate and proximal phalanges. These comparisons potentially indicate a general ape (including humans) pattern of peak pressure location during arboreal posture and locomotion, but data from additional hominoid species are required to verify this hypothesis. The loading pattern similarity is particularly interesting given the curvature and length differences between the phalanges of humans and bonobos (Susman, 1979). This similarity of the human and bonobo patterns may reflect increased flexion at the human distal phalangeal joints when gripping large diameters, which would lead to a decrease in the hand-to-substrate curvature fit, effectively lifting the intermediate phalanges away from the support (Seo and Armstrong, 2008). Whilst the kinematics of the human hand during arboreal locomotion have not been quantified in this study, they should be considered in future work.

The shared pattern of high peak pressure at the third ray during human and bonobo suspension and vertical climbing is also consistent with loading quantified in locomotor behaviors in other primates. The predominant loading of the third ray metacarpal heads in terrestrial palmigrade and digitigrade locomotion (olive baboons: Patel and Wunderlich, 2010), the third ray intermediate phalanges during terrestrial and arboreal knuckle-walking (bonobos: Samuel et al., 2018; chimpanzees: Wunderlich and Jungers, 2009), the third ray proximal palm and distal phalanges during human vertical climbing (this study), and the progression from the proximal phalanges to the proximal palm and distal phalanges with increasing diameter during human suspension (this study) suggests that locomotor categories may be distinguished via distal-proximal loading patterns.

### Implications for interpreting hominin hand morphology

The hand has played a key role in multiple locomotor and manipulative behaviors throughout hominin evolutionary history, but the vast majority of experimental studies have focused on tool-related behaviors (e.g. Key et al., 2019; Key and Dunmore, 2015; Marzke, 1997; Rolian et al., 2011; Williams et al., 2012; Williams-Hatala et al., 2018). Suspension and vertical climbing peak pressure locations on our largest diameter (105 mm) (Fig. 1A,B) show similarities to the loading patterns of Oldowan stone tool knapping, where within the dominant hand the distal phalanges of digits 1-3 experience greater pressures compared to the fourth and fifth digits (Williams et al., 2012; Williams-Hatala et al., 2018). In contrast, the non-dominant hand predominantly loads the first, second, and fifth distal phalanges alongside strong recruitment of the whole fifth digit (Key et al., 2019; Key and Dunmore, 2015). Thus, potential bone functional adaption for either arboreal grasping or tool-related activities maybe challenging to tease apart, particularly within the dominant hand. The current earliest evidence for bipedalism in the hominin fossil record is around 6 mya (Pickford et al., 2002) and possibly earlier (Daver et al., 2022), where hands would have had a reduced, but likely still significant, functional role in locomotion for millions of years at least in some taxa (e.g. Kivell et al., 2015; Prang et al., 2025; Rein et al., 2017). The earliest currently known stone tool technologies appear around 3 mya [Lomekwi: 3.3 mya (Harmand et al., 2015); Oldowan: 2.75 mya (Braun et al., 2025)], but do not become a ‘dominant’ feature of the archaeological record until much later (Barr et al., 2022). However, organic tool use or complex food processing are likely common among all hominins based on comparative observations of organic tool use in extant apes (e.g. Kinani and Zimmerman, 2015; Koops et al., 2015; Luncz et al., 2024). This may indicate a functional overlap in manipulative and arboreal locomotor behaviors for arboreal related morphological characteristics in the hand. Within the hominin fossil record, the presence of curved phalanges together with distal pollical morphology that suggests precision grip abilities in *Orrorin tugenensis* at ∼6 Ma and more recent hominins, alongside greater similarities between modern human and Miocene ape hand proportions than to extant *Pan* or *Pongo* may suggest the exaptation of arboreal-related morphology for manual manipulative abilities in early hominins (Almécija et al., 2010; Almécija et al., 2015; Gommery and Senut, 2006; Senut et al., 2001). However, additional fossil evidence, particularly of African ape ancestors, and detailed biomechanical modeling are needed to test this hypothesis.

A representative sample of the currently available raw pressure data for arboreal locomotion, stone tool manufacture, and stone tool use for key locations in the hand are summarized in Table 3. The mean peak pressures experienced by the hand during arboreal locomotor activities are generally greater than those experienced during stone tool manufacture and use. While no significant differences in raw peak pressure were found between suspension and vertical climbing within bonobos (Samuel et al., 2018), human suspensory raw peak pressure data were generally more than twice the value of that of vertical climbing. Body positioning relative to the hand during an activity (e.g. sitting whilst using a tool versus body mass below the hand during suspension versus body mass roughly parallel to the substrate during climbing) will impact the magnitude of the pressure experienced by the hand. However, species-specific morphological differences, such as hand proportions, limb proportions or kinematics, may counteract some of the effects of differences in body mass positioning. Based on the limited hominid data available (Table 3), the between species difference is most evident in suspension compared to vertical climbing, and within humans the greatest difference between suspensory and vertical climbing pressure magnitudes is observed on the smaller (45 mm) diameter (see Table S2). Thus, use of small diameter supports may have the greatest potential impact on hand morphology. However, more experimental data on hand loading across hominids and across differing substrates are needed. Additional factors, such as the frequency and duration of loading or when in ontogeny loading occurs (e.g. Ruff et al., 2006), also play a role in the behavior-morphology relationship and should be considered in future studies when evaluating functional morphology of the hand and the inferences that can be made about hominin behavioral capabilities.

**Table 3.** Raw peak pressure mean, standard deviation, and range values (where available) for representative examples and key regions (whole hand for locomotion, digits 1 and 3 for stone tool manufacture and use) for human suspension (all diameters combined 433 trials, *n*=14) and vertical climbing (all diameters combined 530 trials, *n*=14), non-human primate arboreal locomotion, stone tool manufacture and stone tool use

Activity	Species	Pressure location(s)	Mean peak pressure (kPa) [s.d.]	Peak pressure range (kPa)
Suspension^a^				
All diameters and activities combined	*H. sapiens*	Peak pressure: See Fig. 1A,B	264.7 [±90.0]	92.5–565.0
Vertical climbing^a^				
All diameters and activities combined	*H. sapiens*	Peak pressure: See Fig. 1A,B	103.5 [±34.8]	30.0–265.0
Non-human primate arboreal locomotion^b^				
Vertical locomotion 120 mm diameter^b^	*P. paniscus*	Palm	142.0 [±12.9]	
		Fingers 2-5	103.3 [±7.6]	
		Thumb	30.0 [±3.4]	
Suspension 120 mm diameter^b^	*P. paniscus*	Palm	129.7 [±12.6]	
		Fingers 2-5	99.7 [±15.7]	
Knuckle-walking 120 mm diameter^b^	*P. paniscus*	Fingers (IPs)	233.6 [±24.2]	
Stone tool manufacture^c,d,e,f^				
Oldowan bifacial chopper: whole sequence^c^	*H. sapiens*	Digit 1	83.9 [±65.0]	15.1–191.1
		Digit 3	132.8 [±75.2]	39.8–214.0
Early Acheulean handaxe (non-dominant hand)^d^	*H. sapiens*	Digit 1	50.5 [±25.9]	15.0–197.5
		Digit 3	40.2 [±24.4]	15.0–192.5
Core securing, knapping floor (non-dominant hand)^e^	*H. sapiens*	DP1	104.0 [±60.3]	38.5–217.3
		DP3	29.6 [±21.7]	2.8–63.3
Core repositioning, knapping floor (non-dominant hand)^e^	*H. sapiens*	DP1	137.9 [±69.3]	66.0–272.3
		DP3	73.1 [±43.2]	17.9–138.9
Flake production^f^	*H. sapiens*	Digit 1	79.2 [±29.9]	
		Digit 3	54.5 [±22.6]	
Stone tool use^f^				
Nut cracking (macadamia)^f^	*H. sapiens*	Digit 1	54.6 [±23.8]	
		Digit 3	40.7 [±18.9]	
Percussive bone marrow extraction^f^	*H. sapiens*	Digit 1	77.7 [±35.3]	
		Digit 3	59.8 [±35.7]	
Slicing meat (medium flake)^f^	*H. sapiens*	Digit 1	58.5 [±27.7]	
		Digit 3	46.0 [±28.8]	
Slicing meat (large handaxe)^f^	*H. sapiens*	Digit 1	66.6 [±27.3]	
		Digit 3	38.1 [±19.3]	

Data from: ^a^ This study, ^b^Samuel et al. (2018) table 2, ^c^Williams et al. (2012), table 2, values in the table below are calculated from the provided individual subject mean peak pressures, ^d^Key et al. (2019). Supporting data, digit values were calculated as averages of the relevant sensors, 0 values were removed following the analysis protocol of Key et al. (2019), ^e^Key and Dunmore (2015) supplementary table 1, values in the table below are calculated from the provided individual subject mean peak pressures, force (kg) was converted to kPa via a coefficient calculated from the gravitational coefficient and force sensor area, ^f^Williams-Hatala et al. (2018) supplementary table 2.

### Limitations

Given the need to keep the human suspension and vertical climbing activities within ethical and laboratory constraints, they are not exact replications of the arboreal activities that humans (or other apes) would engage in within a natural environment (Kraft et al., 2014). The use of separate, minimally overlapping participant groups prevents intra-individual comparison between substrate orientations and introduces inter-individual variation as a possible confounding variable. Whilst our sample size per support orientation (*n*=14) is within the range of previous stone tool force and pressure hand studies [e.g. Key and Dunmore, 2015 (*n*=8); Williams-Hatala et al., 2018 (*n*=39)], it is still modest and there are the effects of behavioral and inter-individual morphological variation that could be investigated. The varying number of usable trials per participant-activity-diameter combination also necessitated the use of participant mean values for each activity-diameter combination to control for pseudoreplication, limiting the number of data points for comparison. The lack of accounting for participant identity in the multinomial logistic regression analyses may lead to the overestimation of effective sample size. Our experimental designed forced participants to be more constrained in their behaviors to allow consistency across inter-individual comparisons, and thus diversity in hand posture and locomotor movements is not fully captured. Variables such as height of activity above ground and compliance of the support are also important factors that likely impact the normalized peak pressure experienced by the hand. That being said, our chosen variables and results provide a good baseline for investigating the impact of specific arboreal behaviors on the human hand. Furthermore, whilst our human data provide a groundwork for investigating the potential effects of arboreal support size on the hand during hominin evolution, the comparative extant hominoid data are limited (see Samuel et al., 2018). Future studies should investigate pressure experienced by the hand across multiple support sizes in arboreal settings across a wider sample of hominoids (and primates more generally) to allow us to fully understand the implications for interpreting hominin arboreal behavioral capabilities from hand morphology.

## Conclusion

Here we presented the first investigation of the effect of support size and orientation on the normalized peak pressure experienced by the human hand during arboreal activities. Thumb position (adducted or abducted) had only minimal impact on normalized peak pressure and was constrained by support orientation (vertical) and smaller support size (45 mm diameter). On the other hand, support diameter had a stronger influence on normalized peak pressure than thumb position. Normalized peak pressure trends between support diameter sizes followed patterns observed in the ergonomic literature, decreasing as the support size increases from the optimal diameter (e.g. Kong and Lowe, 2005; Seo et al., 2007), for both horizontal and vertical support orientations. However, the effect of the ratio between hand length and support size should be examined further. The third ray experiences high loading frequencies for both suspension and vertical climbing, but horizontal and vertical support use could be distinguished via distal-proximal loading patterns. Future studies should investigate arboreal hand pressures in a larger sample of hominoids. Additional variables, such as support compliance, would also provide further insight into support use choices and their impacts on hand morphology in extant primates and extinct hominins.

## MATERIALS AND METHODS

Data were collected at the School of Sport and Exercise Sciences, University of Kent, Chatham Maritime, UK, in collaboration with Skeletal Biology Research Centre, in the former School of Anthropology and Conservation at University of Kent, Canterbury, UK.

### Participants

14 adults (*n*=6 females, 8 males) aged 24-42 years took part in the suspension activities and 14 adults (*n*=8 females, 6 males) aged 20-44 years participated in the vertical climbing activities. Only two participants (1 female, 1 male) were able to participate in both the suspension and vertical climbing experiments because data were collected across two separate trial periods. All participants were volunteers with no known hand injuries or orthopedic impairments in the year prior to their participation. Participants signed informed consent forms prior to data collection. Ethical approval and risk assessment were obtained from the University of Kent.

### Experimental apparatus

**Fig. 2.**
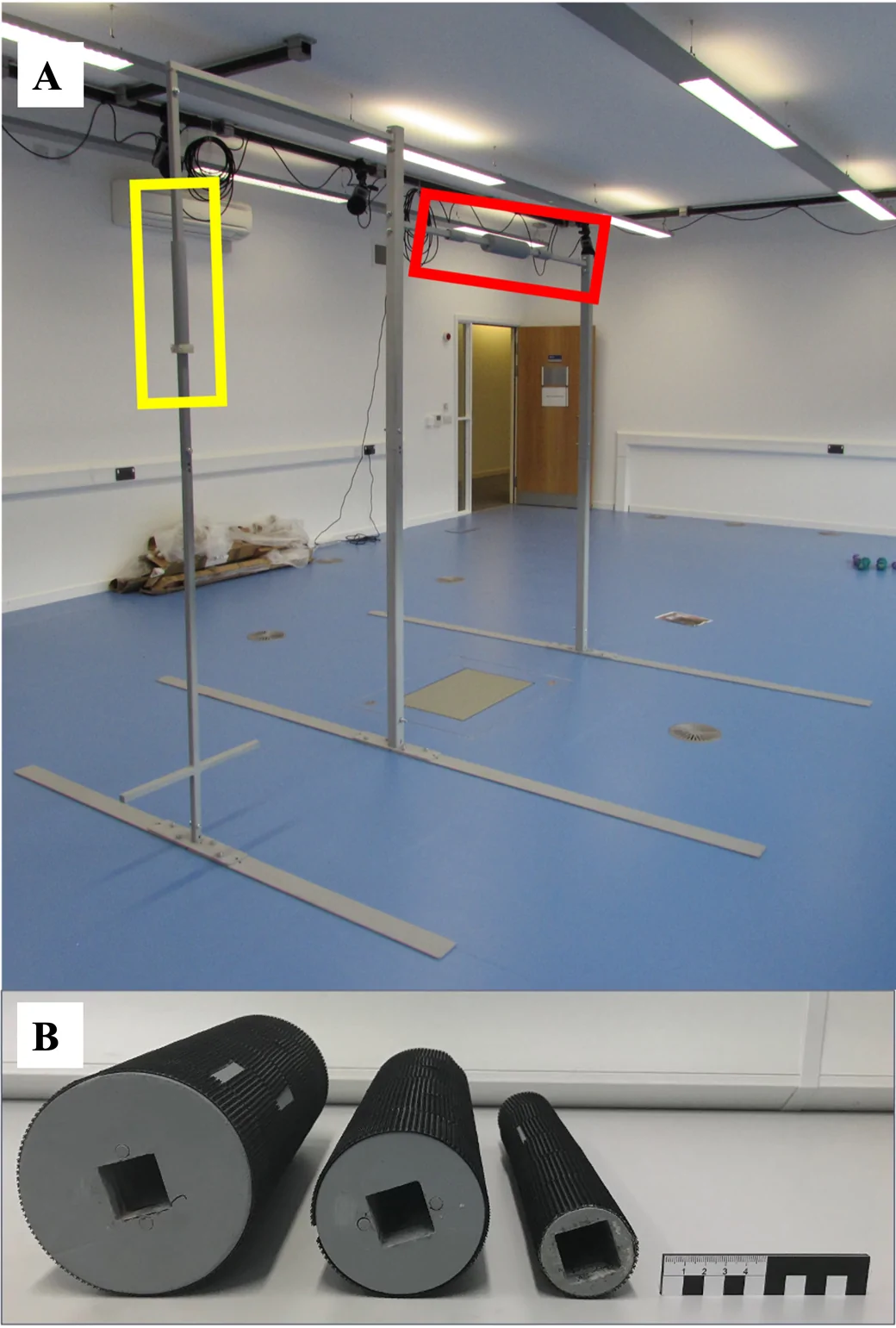
**Experimental apparatus and interchangeable instrumented sleeves (diameters: 105 mm, 80 mm, and 45 mm) manufactured by the University of Kent School of Architecture.** (A) Custom-built climbing frame designed with and built by Sports Equip (http://www.sportsequip.co.uk). Red horizontal box: suspension activity section, yellow vertical box: vertical climbing activity section. (B) A pressure mat was applied to the outside of the sleeve and the Velcro sleeve coating prevented the pressure mat from moving during the activities.

A custom-built climbing frame (Sports Equip, http://www.sportsequip.co.uk) was used for all suspension and vertical climbing activities (Fig. 2A). A gray matte finish was used to minimize reflections on the metal frame during motion capture data collection. Three plywood sleeves of 45 mm, 80 mm, and 105 mm diameters and 300 mm in length were manufactured by the University of Kent School of Architecture and used for both suspension and vertical climbing activities (Fig. 2B). Each sleeve was coated in a gray matte finish. To prevent rotation and ensure the structural security of the equipment, the plywood sleeves were inserted onto a 25×25 mm square vertical (climbing) or horizontal (suspension) bar. A second gray matte-coated plywood cylindrical sleeve of 45 mm diameter and 100 mm length was positioned after the 300 mm length sleeve to provide an additional handhold during the dynamic activity.

To prevent contact with the floor whilst ensuring participant safety during suspension activities the horizontal bar was set at a height of 2.1 m with exercise padding placed below. The 300 mm length sleeve was positioned centrally on the bar. The vertical climbing bar was 2.4 m in height with the cylindrical sleeve positioned at 1.5 m above the ground. Footrests were provided near the base of the vertical climbing bar.

### Pressure data

A rubber coated flexible Novel ® S2119 pressure mat (Novel GmbH, Munich, Germany) with 512 1×1 cm sensors (32 columns, 16 rows) was used to measure pressure. The pressure mat was attached to a battery pack powered Pliance®-xf-32 analyser box (Novel GmbH, Munich, Germany). A data collection rate of 35 Hz was used with a Bluetooth connection used to transmit data to a computer with Pliance ®-xf-32 Recorder software (version 24.3.5; Novel GmbH, Munich, Germany). The Pedar side wireless box was connected to the Pliance ®-xf-32 analyser box via a fiberoptic cable. The Qualisys (Qualisys AB, Goteburg, Sweden) motion capture system was connected to the operator side wireless sync box by a BNC adapted cable. The combination of synchronized pressure and kinematic systems allowed the identification of precise locations of peak pressure in the hand. A pressure mat calibration range of 15-600 kPa was used and validated as suitable via pilot data collection for static and dynamic activities.

To prevent movement during activities, Velcro^®^ was used to secure the pressure mat to the interchangeable diameters. The combined Velcro^®^ (5 mm) and pressure mat (4 mm) thickness resulted in an additional 18 mm on each diameter. Given the deformability of the pressure mat the exact thickness between the hand and the diameter during the trials is unknown, thus we refer to the non-deformable diameter measurements but acknowledge that the true diameters during the activities were marginally larger. To maximize sensor surface area for grasping the pressure mat was positioned widthways on the 45 mm diameter but lengthways on the 80 mm and 105 mm diameters. Some pressure mat overlap occurred on the 80 mm diameter, so care was taken to position the overlap away from the active grasping zone and trials with the hand positioned in the overlap were excluded from analysis. Slight ‘noise’ (low level sensor activation away from the hand) was present for some trials, but trials with high ‘noise’ during grasping were excluded. To provide a comparable baseline the pressure mat was zeroed prior to each trial. No pressure mat was applied to the aforementioned additional handhold.

Pliance-x-32 software (Novel, GmbH, Munich, Germany) was used to process pressure data. Thumb adducted trials were considered usable if the hand was not over the edge of the mat or if it touched the edge of the mat with low pressure (∼15-60 kPa) values in that area. Thumb abducted trials were also considered usable if the whole hand was clearly placed within the mat's bounds for the specific diameter or the palm and/or thumb were just on the mat's edge but exhibited low pressure values. For some participants the hand with the thumb abducted did not completely fit in the bounds of the mat. In these cases, usable trials were classified as the palm or the thumb being within the mat's bounds for the specific diameter. Peak pressure values were noted for each trial and normalized by dividing by the calculated value of hand area (third ray x palm width) divided by body mass.

### Kinematic data

To quantify the position of the dorsal aspect of each participant's first and third ray and wrist, 20 4 mm retroreflective markers were attached using double-sided tape. Single markers were used at the head and base of anatomical elements with one degree of freedom, whereas triads were used to define anatomical elements associated with joints with multiple degrees of freedom. Both the first and third rays were defined using eight markers, one marker was positioned at the head of the fourth metacarpal, and three markers (proximal ulnar, and the radial and ulnar styloid processes) defined the wrist. To reduce excess marker movement during metacarpophalangeal joint flexion, the markers at the third and fourth metacarpal heads were positioned just proximal to the metacarpal heads.

Qualisys (Qualisys AB Goteburg, Sweden) motion capture technology and 11 Oqus cameras (Qualisys AB, Goteburg, Sweden) were used to collect retroreflective marker data, which were labelled and tracked via Qualisys Track Manager (version 2.15; Qualisys AB, Goteburg, Sweden). The 11 cameras were positioned on a ceiling runner or adjustable tripods surrounding the climbing apparatus, with specific camera positions moved to optimize coverage for suspension versus vertical climbing activities. Camera settings (e.g. focus) were optimized prior to calibration and data collection. Each retroreflective marker on the participant's hand was required to be visible to a minimum of three cameras during all trials. A 501.5 mm wand and an L-frame placed in the designated activity area and visible to all cameras were used to calibrate the system for the respective suspension and vertical climbing setups.

Synchronized starting of the Novel Pliance pressure and Qualisys kinematic systems permitted identification of the corresponding pressure and kinematic peak pressure frames. Qualisys automatically ran for 15 s whereas Novel Pliance was stopped manually. Activity length was approximately 5 s for all activities. Qualisys sampled at a frequency of 105 Hz and Novel Pliance sampled at a frequency of 35 Hz, providing a frame ratio of 3:1. Spatial alignment of the pressure data and the marker data was achieved via markers placed in known locations on the margins of the pressure mat.

**Fig. 3.**
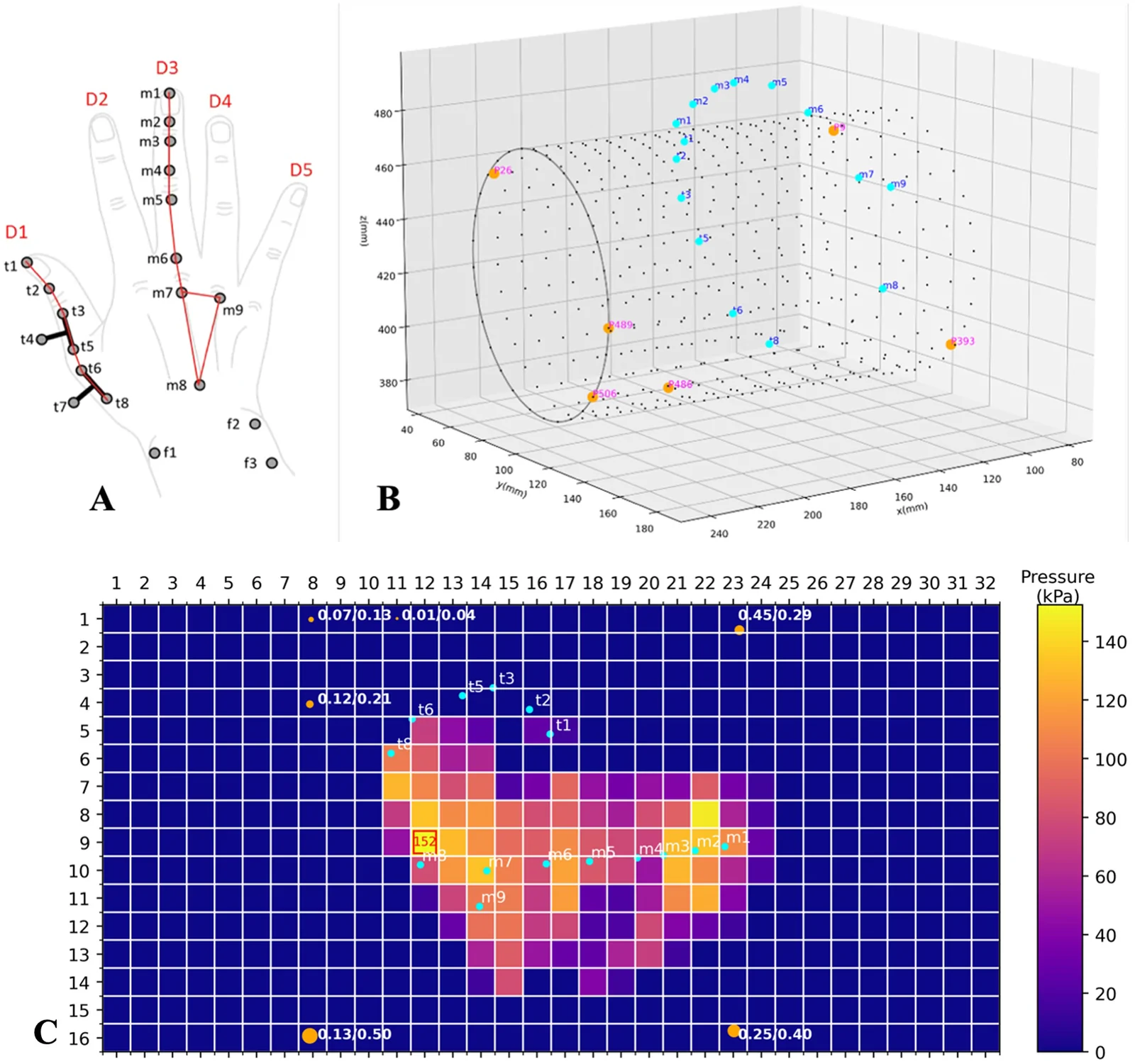
**Retroreflective marker positions on the hand, cylinder model mapping the 3D kinematic data to the 2D pressure mat data for a suspension 80 mm diameter static thumb adducted trial, and Kinematic-to-Pressure mapping output for suspension 80 mm diameter static thumb adducted trial.** (A) Positions of the retroreflective markers on the first (t1-8) and third (m1-8) rays, fourth metacarpal head (m9), and wrist (f1-3). Digit number indicated via D1-5. (B) Retroreflective markers on the pressure mat (orange dots) were used to fit the cylinder model and subsequently calculate the pressure mat sensor positions (black dots). The retroreflective markers on the hand are indicated via labeled cyan dots. (C) Pressure mat sensors (1 cm by 1 cm) are indicated by squares. Pressure mat retroreflective marker (orange circle) size is determined by the amount of uncertainty in the respective marker's measurement. The noted values in white for each pressure mat marker are 1) the distance (cm) from the center of their assigned pressure mat sensor, and 2) the respective marker measurement uncertainty. The hand retroreflective markers (first ray: t1-t8, third ray: m1-m8, fourth metacarpal head: m9) are indicated by cyan circles. The varying color map (see values on scale on the righthand side of the figure) displays pressure values (kPa) within the specific peak pressure frame. The location of peak pressure (kPa) is indicated by the red outlined square and associated value within the square.

Qualisys Track Manager was used to process kinematic data. Gap filled trajectories were removed to prevent marker path assumptions and only measured trajectories were analyzed. Filters were not used in the data collection, processing, or subsequent Kinematic-to-Pressure mapping analysis. A usable kinematic file had the circle fitting markers for the Kinematic-to-Pressure mapping process present alongside the markers on the first and third rays and wrist (t1-3, t5-6, t8, m1-9, f1-3, see Fig. 3A) during the frames corresponding to the peak pressure frames in the associated pressure data file. After processing, kinematic files were converted to .mat file format for the mapping analysis.

### Participant biometric data

Participant body mass was measured whilst wearing shoes, as they were worn during the experiment, whereas height was measured without shoes. Participants' age, sex, and dominant hand were noted, with length of the third ray and palm width measured in ImageJ (Version 2.0.0-rc-43/1.51o: Schneider et al., 2012) from digital scans including a scale bar. Body mass data were used alongside hand area data to normalize the pressure data.

### Experimental design

Participants undertook three activities for both suspension and vertical climbing categories: (1) static position with adducted thumb, (2) static position with abducted thumb, and (3) dynamic suspension/vertical climbing with an adducted or abducted thumb based on the participant's preference. All participants were given the same hand positioning instructions but were able to position their thumb comfortably within the assigned activity category. All three activities were completed before moving on to the next diameter. The three diameters were presented to the participants in a randomized order. A minimum of three trials were run per activity-diameter combination to ensure usable data. Each participant was able to briefly familiarize themselves with each activity and diameter before active data collection commenced.

#### Static thumb adducted and abducted

**Fig. 4.**
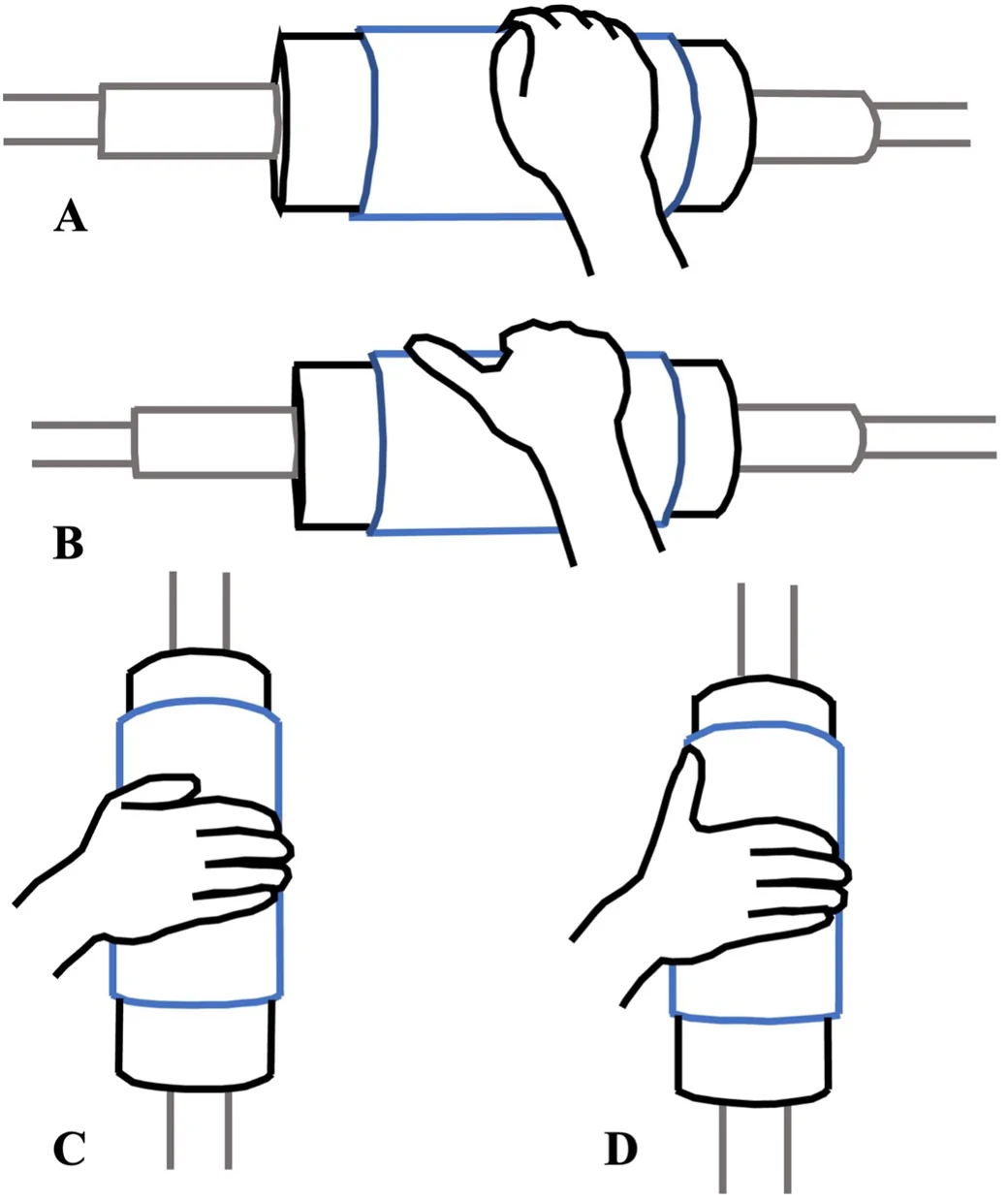
**Suspension (A,B) and vertical climbing (C,D) static activities' hand positioning relative to the pressure mat.** (A) Static thumb adducted suspension, (B) static thumb abducted suspension, (C) static thumb adducted vertical climbing, and (D) static thumb abducted vertical climbing.

For the static suspension trials, participants stood below the suspension pole with their sagittal plane parallel to the pole. Their dominant hand with either adducted or abducted thumb was positioned near the instrumented sleeve and then placed onto the instrumented sleeve, within the diameter specific bounds of the pressure mat (see Fig. 4A,B). The participants then raised their feet off the floor and suspended from their hand for ∼5 s before lowering their feet and letting go of the instrumented sleeve.

For the vertical climbing trials, participants stood on the footrests of the vertical climbing pole and grasped the instrumented sleeve with their dominant hand and adducted or abducted thumb (see Fig. 4C,D). Participants then held a slightly lent back position for ∼5 s before righting themselves, letting go of the instrumented sleeve, and stepping down on to the floor.

In the aforementioned cases where the participant's hand with abducted thumb did not fit within the diameter specific bounds of the pressure mat, separate trials for the palm and thumb were collected. A Mann–Whitney *U*-test was used to examine differences in normalized peak pressure between these trials and those with the hand within the diameter specific pressure mat bounds.

#### Dynamic activities

**Fig. 5.**
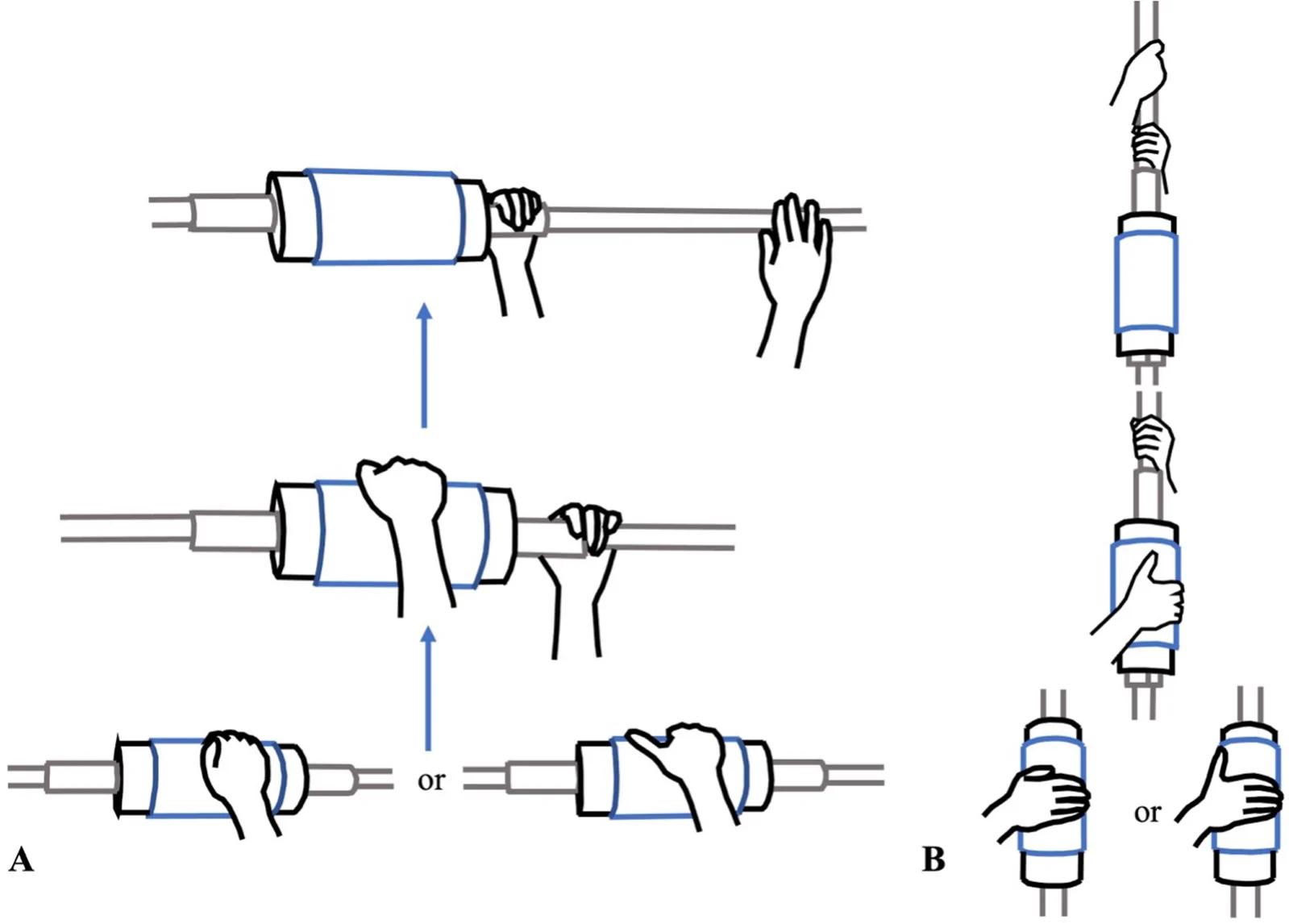
Dynamic suspension (A) and vertical climbing (B) activity steps.

To complete the dynamic suspension activity the participant stood below the suspension pole, with their sagittal plane aligned to the pole, and positioned their dominant hand near, but not on, the instrumented portion of the pole. Then they grasped the pressure mat, raised their feet off the floor, and swung themselves along the pole to grasp the additional handhold with their non-dominant hand. Their dominant hand then released instrumented sleeve and they lowered their feet to the floor before fully letting go of the suspension pole. See Fig. 5A.

To complete the dynamic vertical climbing the participant stepped onto the footrest and with their dominant hand grasped the instrumented sleeve and grasped above the diameter with their non-dominant hand. They then pulled themselves up slightly and released the diameter to grasp further up the pole with their dominant hand. See Fig. 5B. The participant then lowered themselves down and stepped off the footrests onto the floor.

### Kinematic-to-Pressure mapping

To map 3D kinematic data (see Fig. 3B) onto the 2D pressure data (see Fig. 3C) we developed a Kinematic-to-Pressure mapping model written in Python (S.-C.L.: code and initial method, H.Y. and V.A.L.: updated code). Usable pairs of kinematic (.mat) and pressure (ASCII) data files were imported into the model. Peak pressure frames were identified in the ASCII file and the corresponding frames were then identified in the kinematic data file (.mat). Given the 3:1 kinematic to pressure frame ratio, the first of every three kinematic frames were selected as it had the closest proximity to the peak pressure moment. A cylinder model using the known positions of six retroreflective markers on the pressure mat was used to map the 3D kinematic data onto the 2D pressure data. Three of these markers were used as reference markers to form a circle (P26, P489, and P506 in Fig. 3B). The cylinder model was defined using seven parameters: the center (X0, Y0, Z0) of the circle, the circle's radius (R0), and the orientation angles (theta, phi, gamma) that describe the cylinder's rotation and orientation. A weighted least squares fitting was applied as the uncertainties and location (3D coordinates) for the six pressure mat markers were known. A minimum of three markers was required for the mapping, but all six of the pressure mat markers were used where possible. Marker specific uncertainties determined marker weights, thus markers with smaller uncertainties had higher weights. After the cylinder model was fitted, it was used to calculate the closest locations on the pressure mat to the markers on the participant's hand (cyan dots in Fig. 3B,C).

### Validation of Kinematic-to-Pressure mapping method

The lack of clearly identifiable features per sensor on the pressure mat exterior and the visually assessed central position on the sensor for each pressure mat marker for each diameter may have introduced slight error into the mapping analyses. Thus, the accuracy of the Kinematic-to-Pressure mapping method was verified via a validation test for each of the diameters.

A participant, with retroreflective markers on their first and third rays and wrist and their feet in contact with the floor, lightly positioned their hand on the instrumented sleeve. Pressure was then sequentially applied at the distal phalanges for digits I-V. The resulting pressure and kinematic files were mapped via the Kinematic-to-Pressure mapping program. The markers on the first and third rays corresponded to the respective pressure sensor activation and the pressure sensor activation for digits II, IV-V had logical placement relative to the first and third positions. Thus, the Kinematic-to-Pressure mapping program's accuracy level was deemed appropriate.

### Location identification

Location identification was based on the method of Borel et al. (2017) and separated into radial-ulnar and proximal-distal analyses. Rays II-V were divided into distal, intermediate, and proximal phalanges, and metacarpal head and base. Ray I was divided into distal and proximal phalanges, and metacarpal head and base. A number was designated for each location. Within the radial-ulnar analysis all locations were grouped by ray (I-V) and compared at the level of the ray (see Fig. S2A). For the proximal-distal analysis, the locations were grouped by type (e.g. distal phalanges) for all rays combined and compared to the other all-ray location types (see Fig. S2B). If a trial contained peak pressure locations at multiple rays or all-ray location types, then a new number was given to each new combination (see Fig. S2A,B).

### Statistical analyses

To account for differences in absolute hand and body size across participants, peak pressure values were normalized by dividing by the calculated value of hand area (third ray x palm width) divided by body mass. Non-parametric tests were used as Shapiro-Wilks tests determined that the suspension and vertical climbing pressure data were not normally distributed. Due to varying numbers of usable trials per participant, a mean normalized peak pressure value per participant for each activity-diameter combination was calculated, resulting in one value per participant, per activity, per diameter where data were available (see Table S12 for mean normalized peak pressure and raw data sample sizes). This reduced the overall size of the dataset but allowed us to control for intraindividual variation within our analyses. These mean normalized participant specific activity-diameter values were used for all subsequent calculations and are henceforth referred to as normalized peak pressure values.

Suspension and vertical climbing data were analyzed separately. For each diameter, differences in normalized peak pressure between static thumb adducted and abducted conditions were assessed by Wilcoxon Signed Rank test with *P*-values adjusted across the three diameter comparisons using the Benjamini and Hochberg (BH) method. A mean static activity value per participant was calculated if no difference was found between the static thumb adducted and abducted conditions. Mean static activity and dynamic activity values were compared via Wilcoxon Signed Rank test and *P*-values were adjusted with the BH method to account for comparisons on multiple diameters. Sample sizes varied depending on data quality (see Table S12).

If there was no difference found between the static thumb adducted and abducted positions and the dynamic and mean static values, a mean normalized peak pressure value for all activities on each diameter was calculated and compared between the three diameters using a Friedman's test. Only data for participants that had available data for all three activities on a diameter were used. Mean combined activity values per participant for each diameter were calculated using the aforementioned mean normalized peak pressure values for each participant-activity-diameter combination to control for issues of pseudoreplication. Further Friedman's tests were used to investigate within diameter-among activity and within activity-among diameter differences in normalized peak pressure with *P*-values adjusted via the BH method for the comparisons between the three activities or diameters, respectively. Where appropriate Nemenyi post-hoc tests with single step *P*-value adjustments were used. These comparisons only included participants with the full set of required data (see Table S12).

Normalized peak pressure variance within diameter-among activity and within activity-among diameter was investigated using Brown Forsythe tests for equality of variance with *P*-values adjusted via the BH method. The same data subsets were used for the Brown-Forsythe tests and the previously described Friedman's tests for within diameter-among activity and within activity-among diameter comparisons. All available data were used to calculate the coefficient of variation (using the mean) of normalized peak pressure for each activity-diameter combination.

Location frequency and mode location were calculated for each activity-diameter combination for suspension and vertical climbing respectively using all usable trials (suspension: 433 trials, vertical climbing: 530 trials), which includes multiple trials per participant, per activity-diameter combination. Peak pressure location for each activity-diameter combination was then analyzed using multinomial logistic regressions and the relative risk ratios were calculated. Whilst the activity and diameter variables were included in the multinomial logistic regressions, it was not possible to account for participant identity in the analysis. To reduce noise, only locations that occurred at 10.0% or greater frequency for each activity-diameter combination were included in the analysis. Reference categories were the most frequent category within peak pressure location (radial-ulnar or distal-proximal), diameter, and activity.

R (version 4.4.0: R Core Team, 2024) and RStudio (version 2024.4.1.748: Posit team 2024) were used for statistical analysis with statistical significance set at 0.05. To control for false discovery rate where necessary *P*-values were adjusted via the BH method, unless otherwise stated.

## Supplementary Material

10.1242/biolopen.062562_sup1 Supplementary information
