# Supplementary material for: Peak pressure experienced by the human hand during suspension and vertical climbing
Source: Biol Open. 2026 Aug 20;15(8):bio062562. doi: 10.1242/bio.062562 (PMC13552479; doi:10.1242/bio.062562)
Supplement: Supplementary file 1 [file biolopen-15-062562-s1.pdf]

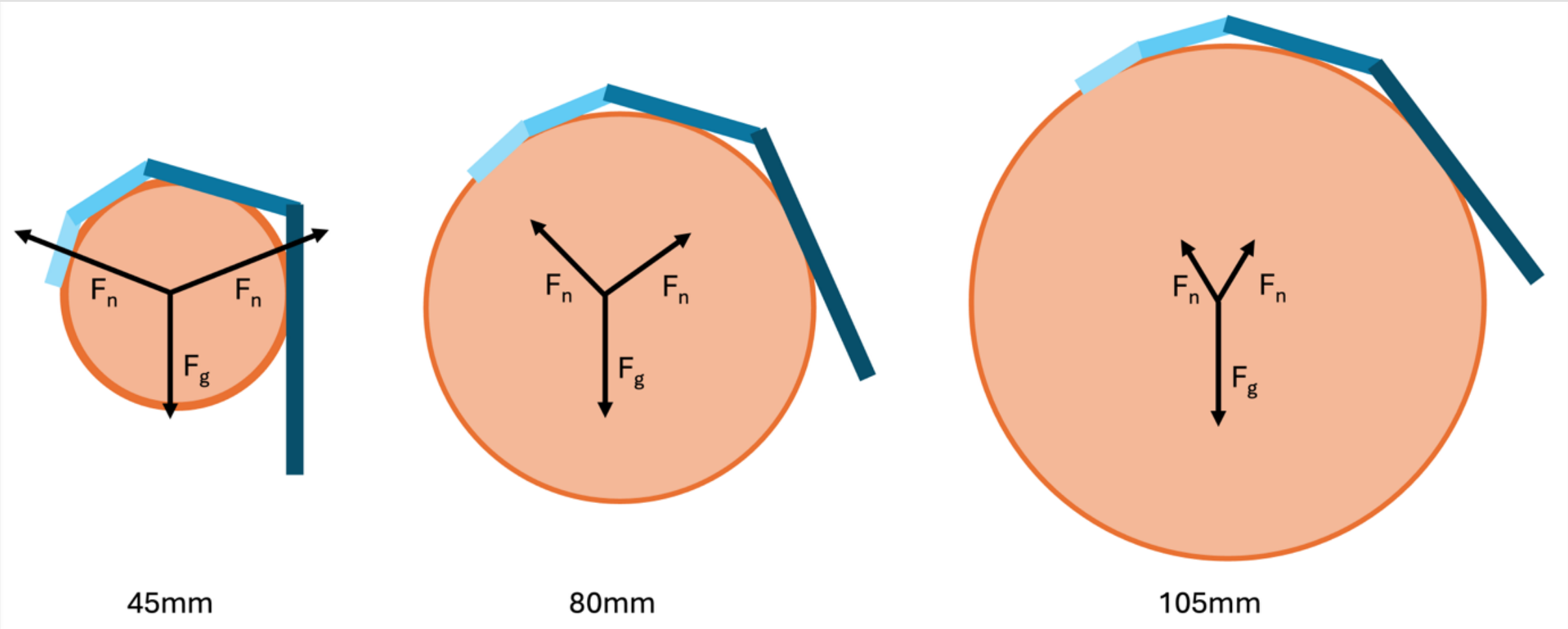

Fig. S1. Free body diagrams with scaled vectors of forces acting on the hand during suspension on support diameters of 45mm, 80mm, and 105mm. F_g: Gravity, F_n: Normal force. All vectors are moved to the center of each diameter for the purpose of illustration. Hand anatomical segments are defined by color gradient, lightest: Distal phalanx, followed by intermediate phalanx and proximal phalanx, darkest: Metacarpals. The wrist is not depicted as its positioning was not quantified in this study. Grasping forces of the hand are not shown as contact area between the hand and support may vary with diameter size and those data were not collected as part of this specific study.

Biology Open • Supplementary information

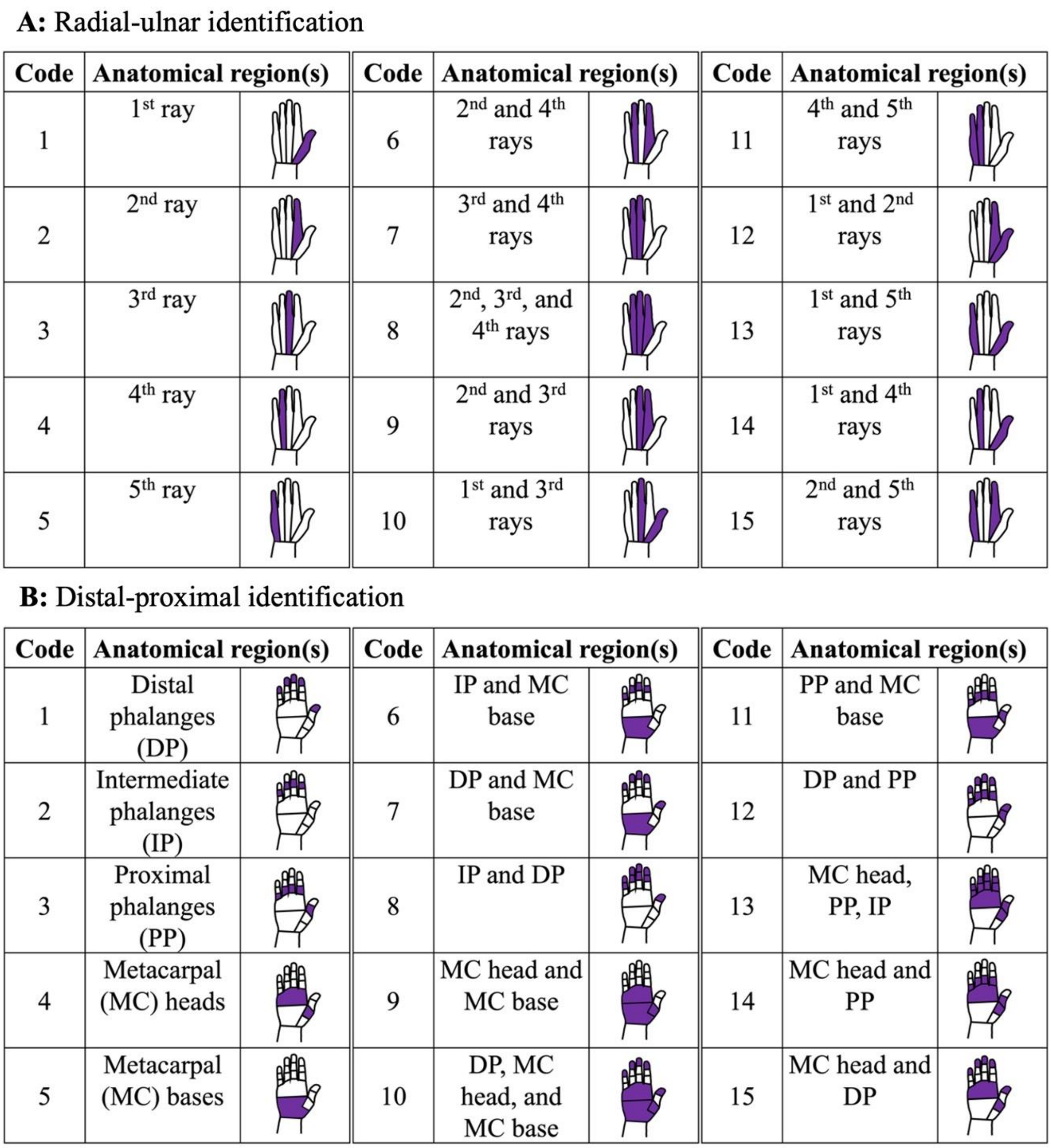

A: Radial-ulnar identification

Code	Anatomical region(s)	Code	Anatomical region(s)	Code	Anatomical region(s)
1	1st ray	6	2nd and 4th rays	11	4th and 5th rays
2	2nd ray	7	3rd and 4th rays	12	1st and 2nd rays
3	3rd ray	8	2nd, 3rd, and 4th rays	13	1st and 5th rays
4	4th ray	9	2nd and 3rd rays	14	1st and 4th rays
5	5th ray	10	1st and 3rd rays	15	2nd and 5th rays

B: Distal-proximal identification

Code	Anatomical region(s)	Code	Anatomical region(s)	Code	Anatomical region(s)
1	Distal phalanges (DP)	6	IP and MC base	11	PP and MC base
2	Intermediate phalanges (IP)	7	DP and MC base	12	DP and PP
3	Proximal phalanges (PP)	8	IP and DP	13	MC head, PP, IP
4	Metacarpal (MC) heads	9	MC head and MC base	14	MC head and PP
5	Metacarpal (MC) bases	10	DP, MC head, and MC base	15	MC head and DP

Fig. S2. Peak pressure location code classifications for radial-ulnar and distal-proximal categories. A) Radial-ulnar peak pressure location categories. B) Distal-proximal peak pressure location categories. Categories 1-5: Single (A) ray or (B) anatomical region locations of peak pressure. Categories 6-15: Peak pressure occurring on multiple (A) rays or (B) anatomical regions within a single trial. Purple hand regions indicated indicate peak pressure location.

Biology Open • Supplementary information

Table S1. Suspension (n = 14) and vertical climbing (n = 14) participant sex, body mass (kg), and hand length (mm).

Participant	Sex	Body mass (kg)	Hand length (mm)
**Suspension**			
**A**	Male	84.0	193.8
**B**	Female	57.0	184.2
**C**	Female	69.0	178.8
**D**	Male	69.0	199.1
**E**	Male	94.0	187.7
**F**	Male	72.0	188.2
**G**	Male	72.0	190.5
**H**	Male	97.5	200.9
**I**	Female	66.5	173.9
**J**	Male	92.0	189.3
**K**	Female	60.0	171.6
**L**	Female	62.0	169.8
**M**	Male	80.0	195.7
**N**	Female	55.5	173.2
**Vertical climbing**			
**O**	Female	55.0	184.2
**P**	Male	99.0	187.7
**Q**	Female	47.5	168.6
**R**	Male	69.0	181.2
**S**	Female	57.0	172.5
**T**	Female	82.0	164.4
**U**	Male	102.0	188.7
**V**	Female	50.5	183.3
**W**	Female	88.0	164.7
**X**	Male	79.0	188.5
**Y**	Male	81.5	196.5
**Z**	Female	60.0	163.6
**AA**	Male	84.5	189.6
**BB**	Female	69.5	171.6

Biology Open • Supplementary information

Table S2. Raw peak pressure mean, standard deviation, and range values for suspension (433 trials, n=14) and vertical climbing (530 trials, n=14).

Diameter (mm)	Activity	Mean peak pressure (kPa) [SD]	Peak pressure range (kPa)
**Suspension**			
**45**	Thumb adducted	345.2 [± 84.2]	187.5 – 537.5
	Thumb abducted	359.9 [± 95.3]	172.5 – 565.0
	Dynamic	316.2 [± 105.1]	155.0 – 527.5
**80**	Thumb adducted	226.9 [± 48.4]	140.0 – 372.5
	Thumb abducted	235.1 [± 62.2]	115.0 – 392.5
	Dynamic	230.8 [± 57.5]	120.0 – 350.0
**105**	Thumb adducted	233.2 [± 59.8]	145.0 – 420.0
	Thumb abducted	211.1 [± 53.0]	115.0 – 355.0
	Dynamic	230.6 [± 68.3]	92.5 – 370.0
**Vertical climbing**			
**45**	Thumb adducted	110.4 [± 29.8]	50.0 – 195.0
	Thumb abducted	124.5 [± 38.5]	57.5 – 220.0
	Dynamic	133.5 [± 48.1]	57.5 – 265.0
**80**	Thumb adducted	88.0 [± 27.8]	30.0 – 190.0
	Thumb abducted	96.9 [± 27.2]	47.5 – 165.0
	Dynamic	108.0 [± 39.6]	40.0 – 192.5
**105**	Thumb adducted	86.9 [± 22.8]	40.0 – 137.5
	Thumb abducted	96.3 [± 26.7]	47.5 – 155.0
	Dynamic	103.0 [± 26.0]	47.5 – 202.5

Table S3. Coefficients of variation, calculated using the mean, for each activity-diameter combination for suspension and vertical climbing.

Activity	Diameter		
	**45mm**	**80mm**	**105mm**
**Suspension**			
**Static thumb adducted**	30.6	28.4	27.8
**Static thumb abducted**	31.3	32.9	30.4
**Dynamic**	37.9	32.5	32.4
**Vertical climbing**			
**Static thumb adducted**	40.0	48.8	40.7
**Static thumb abducted**	44.8	43.2	42.0
**Dynamic**	52.0	52.6	34.9

Suspension

Diameter (mm)	Activity	Radial-ulnar location								
		**1**	**2**	**3**	**4**	**5**	**7**	**9**	**11**	**Total**
**45**	**Static thumb adducted (%)**	0 (0)	7 (13.0)	29 (53.7)	16 (29.6)	1 (1.9)	0 (0)	1 (1.9)	0 (0)	54 (100)
	**Static thumb abducted (%)**	1 (2.1)	2 (4.3)	25 (53.2)	16 (34.0)	2 (4.3)	0 (0)	0 (0)	1 (2.1)	47 (100)
	**Dynamic (%)**	0 (0)	4 (9.8)	22 (53.7)	13 (31.7)	1 (2.4)	1 (2.4)	0 (0)	0 (0)	41 (100)
	**Total (%)**	**1 (0.7)**	**13 (9.2)**	**76 (53.5)**	**45 (31.7)**	**4 (2.8)**	**1 (0.7)**	**1 (0.7)**	**1 (0.7)**	**142 (100)**

Table S4. Radial-ulnar peak pressure location for all 433 suspension trials. Counts of each location frequency for each diameter-activity combination are noted, with the row percentage noted in parentheses. Continued on the next two pages.

Diameter (mm)	Activity	Radial-ulnar location								
		**1**	**2**	**3**	**4**	**5**	**7**	**9**	**11**	**Total**
**80**	**Static thumb adducted (%)**	8 (13.8)	8 (13.8)	33 (56.9)	8 (13.8)	0 (0)	1 (1.7)	0 (0)	0 (0)	58 (100)
	**Static thumb abducted (%)**	1 (1.9)	5 (9.4)	38 (71.7)	8 (15.1)	0 (0)	1 (1.9)	0 (0)	0 (0)	53 (100)
	**Dynamic (%)**	10 (19.6)	16 (31.4)	20 (39.2)	4 (7.8)	0 (0)	0 (0)	1 (2.0)	0 (0)	51 (100)
	**Total (%)**	**19 (11.7)**	**29 (17.9)**	**91 (56.2)**	**20 (12.3)**	**0 (0)**	**2 (1.2)**	**1 (0.6)**	**0 (0)**	**162 (100)**

Table S4. Continued, 80mm.

Diameter (mm)	Activity	Radial-ulnar location								
		**1**	**2**	**3**	**4**	**5**	**7**	**9**	**11**	**Total**
**105**	**Static thumb adducted (%)**	4 (12.1)	12 (36.4)	17 (51.5)	0 (0)	0 (0)	0 (0)	0 (0)	0 (0)	33 (100)
	**Static thumb abducted (%)**	1 (1.8)	12 (21.1)	42 (73.7)	1 (1.8)	1 (1.8)	0 (0)	0 (0)	0 (0)	57 (100)
	**Dynamic (%)**	9 (23.1)	18 (46.2)	11 (28.2)	0 (0)	0 (0)	0 (0)	1 (2.6)	0 (0)	39 (100)
	**Total (%)**	**14 (10.9)**	**42 (32.6)**	**70 (54.3)**	**1 (0.8)**	**1 (0.8)**	**0 (0)**	**1 (0.8)**	**0 (0)**	**129 (100)**

Table S4. Continued, 105mm.

Diameter (mm)	Activity	Distal-proximal location												
		**1**	**2**	**3**	**4**	**5**	**7**	**8**	**9**	**12**	**13**	**14**	**15**	**Total**
**45**	**Static thumb adducted (%)**	4 (7.4)	9 (16.7)	35 (64.8)	5 (9.3)	0 (0)	0 (0)	1 (1.9)	0 (0)	0 (0)	0 (0)	0 (0)	0 (0)	54 (100)
	**Static thumb abducted (%)**	5 (10.6)	6 (12.8)	32 (68.1)	3 (6.4)	0 (0)	0 (0)	0 (0)	0 (0)	0 (0)	1 (2.1)	0 (0)	0 (0)	47 (100)
	**Dynamic (%)**	4 (9.8)	4 (9.8)	29 (70.7)	2 (4.9)	0 (0)	0 (0)	0 (0)	0 (0)	2 (4.9)	0 (0)	0 (0)	0 (0)	41 (100)
	**Total (%)**	**13 (9.2)**	**19 (13.4)**	**96 (67.6)**	**10 (7.0)**	**0 (0)**	**0 (0)**	**1 (0.7)**	**0 (0)**	**2 (1.4)**	**1 (0.7)**	**0 (0)**	**0 (0)**	**142 (100)**

Table S5. Distal-proximal peak pressure location for all 433 suspension trials. Counts of each location frequency for each diameter-activity combination are noted, with the row percentage noted in parentheses. Continued on the next two pages.

Diameter (mm)	Activity	Distal-proximal location												
		1	2	3	4	5	7	8	9	12	13	14	15	Total
**80**	**Static thumb adducted (%)**	14 (24.1)	10 (17.2)	17 (29.3)	12 (20.7)	5 (8.6)	0 (0)	0 (0)	0 (0)	0 (0)	0 (0)	0 (0)	0 (0)	58 (100)
	**Static thumb abducted (%)**	7 (13.2)	10 (18.9)	15 (28.3)	14 (26.4)	6 (11.3)	0 (0)	0 (0)	0 (0)	0 (0)	0 (0)	1 (1.9)	0 (0)	53 (100)
	**Dynamic (%)**	5 (9.8)	2 (3.9)	3 (5.9)	21 (41.2)	18 (35.3)	0 (0)	0 (0)	2 (3.9)	0 (0)	0 (0)	0 (0)	0 (0)	51 (100)
	**Total (%)**	**26 (16.0)**	**22 (13.6)**	**35 (21.6)**	**47 (29.0)**	**29 (17.9)**	**0 (0)**	**0 (0)**	**2 (1.2)**	**0 (0)**	**0 (0)**	**1 (0.6)**	**0 (0)**	**162 (100)**

Table S5. Continued, 80mm.

Diameter (mm)	Activity	Distal-proximal location												
		**1**	**2**	**3**	**4**	**5**	**7**	**8**	**9**	**12**	**13**	**14**	**15**	**Total**
**105**	**Static thumb adducted (%)**	13 (39.4)	2 (6.1)	5 (15.2)	3 (9.1)	10 (30.3)	0 (0)	0 (0)	0 (0)	0 (0)	0 (0)	0 (0)	0 (0)	33 (100)
	**Static thumb abducted (%)**	25 (43.9)	4 (7.0)	9 (15.8)	7 (12.3)	11 (19.3)	0 (0)	0 (0)	0 (0)	0 (0)	0 (0)	1 (1.8)	0 (0)	57 (100)
	**Dynamic (%)**	9 (23.1)	4 (10.3)	3 (7.7)	7 (17.9)	13 (33.3)	1 (2.6)	0 (0)	1 (2.6)	0 (0)	0 (0)	0 (0)	1 (2.6)	39 (100)
	**Total (%)**	**47 (36.4)**	**10 (7.8)**	**17 (13.2)**	**17 (13.2)**	**34 (26.4)**	**1 (0.8)**	**0 (0)**	**1 (0.8)**	**0 (0)**	**0 (0)**	**1 (0.8)**	**1 (0.8)**	**129 (100)**

Table S5. Continued, 105mm.

Vertical climbing

Diameter (mm)	Activity	Radial-ulnar location														
		**1**	**2**	**3**	**4**	**5**	**6**	**7**	**8**	**9**	**10**	**11**	**12**	**14**	**15**	**Total**
**45**	**Static thumb adducted (%)**	3 (5.5)	5 (9.1)	27 (49.1)	18 (32.7)	1 (1.8)	1 (1.8)	0 (0)	0 (0)	0 (0)	0 (0)	0 (0)	0 (0)	0 (0)	0 (0)	55 (100)
	**Static thumb abducted (%)**	0 (0)	4 (7.7)	18 (34.6)	26 (50.0)	0 (0)	1 (1.9)	3 (5.8)	0 (0)	0 (0)	0 (0)	0 (0)	0 (0)	0 (0)	0 (0)	52 (100)
	**Dynamic (%)**	5 (9.8)	5 (9.8)	18 (35.3)	19 (37.3)	2 (3.9)	0 (0)	1 (2.0)	1 (2.0)	0 (0)	0 (0)	0 (0)	0 (0)	0 (0)	0 (0)	51 (100)
	**Total (%)**	**8 (5.1)**	**14 (8.9)**	**63 (39.9)**	**63 (39.9)**	**3 (1.9)**	**2 (1.3)**	**4 (2.5)**	**1 (0.6)**	**0 (0)**	**0 (0)**	**0 (0)**	**0 (0)**	**0 (0)**	**0 (0)**	**158 (100)**

Table S6. Radial-ulnar peak pressure location for all 530 vertical climbing trials. Counts of each location frequency for each diameter-activity combination are noted, with the row percentage noted in parentheses. Continued on the next two pages.

Diameter (mm)	Activity	Radial-ulnar location														
		**1**	**2**	**3**	**4**	**5**	**6**	**7**	**8**	**9**	**10**	**11**	**12**	**14**	**15**	**Total**
**80**	**Static thumb adducted (%)**	5 (6.8)	19 (26.0)	34 (46.6)	8 (11.0)	0 (0)	0 (0)	0 (0)	1 (1.4)	4 (5.5)	0 (0)	1 (1.4)	1 (1.4)	0 (0)	0 (0)	73 (100)
	**Static thumb abducted (%)**	5 (8.2)	15 (24.6)	20 (32.8)	13 (21.3)	3 (4.9)	0 (0)	1 (1.6)	0 (0)	2 (3.3)	1 (1.6)	0 (0)	0 (0)	1 (1.6)	0 (0)	61 (100)
	**Dynamic (%)**	7 (16.3)	10 (23.3)	11 (25.6)	12 (27.9)	2 (4.7)	0 (0)	0 (0)	1 (2.3)	0 (0)	0 (0)	0 (0)	0 (0)	0 (0)	0 (0)	43 (100)
	**Total (%)**	**17 (9.6)**	**44 (24.9)**	**65 (36.7)**	**33 (18.6)**	**5 (2.8)**	**0 (0)**	**1 (0.6)**	**2 (1.1)**	**6 (3.4)**	**1 (0.6)**	**1 (0.6)**	**1 (0.6)**	**1 (0.6)**	**0 (0)**	**177 (100)**

Table S6. Continued, 80mm.

Diameter (mm)	Activity	Radial-ulnar location														
		**1**	**2**	**3**	**4**	**5**	**6**	**7**	**8**	**9**	**10**	**11**	**12**	**14**	**15**	**Total**
**105**	**Static thumb adducted (%)**	6 (8.7)	15 (21.7)	38 (55.1)	4 (5.8)	0 (0)	0 (0)	1 (1.4)	0 (0)	4 (5.8)	1 (1.4)	0 (0)	0 (0)	0 (0)	0 (0)	69 (100)
	**Static thumb abducted (%)**	10 (15.4)	9 (13.8)	34 (52.3)	6 (9.2)	0 (0)	0 (0)	3 (4.6)	0 (0)	2 (3.1)	0 (0)	0 (0)	0 (0)	0 (0)	1 (1.5)	65 (100)
	**Dynamic (%)**	7 (11.5)	14 (23.0)	34 (55.7)	4 (6.6)	1 (1.6)	0 (0)	0 (0)	0 (0)	1 (1.6)	0 (0)	0 (0)	0 (0)	0 (0)	0 (0)	61 (100)
	**Total (%)**	**23 (11.8)**	**38 (19.5)**	**106 (54.4)**	**14 (7.2)**	**1 (0.5)**	**0 (0)**	**4 (2.1)**	**0 (0)**	**7 (3.6)**	**1 (0.5)**	**0 (0)**	**0 (0)**	**0 (0)**	**1 (0.5)**	**195 (100)**

Table S6. Continued, 105mm.

Diameter (mm)	Activity	Distal-proximal location											
		**1**	**2**	**3**	**4**	**5**	**6**	**7**	**8**	**9**	**10**	**11**	**Total**
**45**	**Static thumb adducted (%)**	15 (27.3)	25 (45.5)	3 (5.5)	2 (3.6)	9 (16.4)	0 (0)	0 (0)	0 (0)	1 (1.8)	0 (0)	0 (0)	55 (100)
	**Static thumb abducted (%)**	8 (15.4)	11 (21.2)	3 (5.8)	0 (0)	25 (48.1)	1 (1.9)	1 (1.9)	1 (1.9)	2 (3.8)	0 (0)	0 (0)	52 (100)
	**Dynamic (%)**	10 (19.6)	14 (27.5)	2 (3.9)	1 (2.0)	23 (45.1)	0 (0)	1 (2.0)	0 (0)	0 (0)	0 (0)	0 (0)	51 (100)
	**Total (%)**	**33 (20.9)**	**50 (31.6)**	**8 (5.1)**	**3 (1.9)**	**57 (36.1)**	**1 (0.6)**	**2 (1.3)**	**1 (0.6)**	**3 (1.9)**	**0 (0)**	**0 (0)**	**158 (100)**

Table S7. Distal-proximal peak pressure location for all 530 vertical climbing trials. Counts of each location frequency for each diameter-activity combination are noted, with the row percentage noted in parentheses. Continued on the next two pages.

Diameter (mm)	Activity	Distal-proximal location											
		**1**	**2**	**3**	**4**	**5**	**6**	**7**	**8**	**9**	**10**	**11**	**Total**
**80**	**Static thumb adducted (%)**	26 (35.6)	9 (12.3)	2 (2.7)	0 (0)	31 (42.5)	1 (1.4)	1 (1.4)	1 (1.4)	1 (1.4)	0 (0)	1 (1.4)	73 (100)
	**Static thumb abducted (%)**	22 (36.1)	11 (18.0)	4 (6.6)	1 (1.6)	18 (29.5)	1 (1.6)	2 (3.3)	1 (1.6)	0 (0)	1 (1.6)	0 (0)	61 (100)
	**Dynamic (%)**	22 (51.2)	6 (14.0)	0 (0)	2 (4.7)	11 (25.6)	0 (0)	1 (2.3)	1 (2.3)	0 (0)	0 (0)	0 (0)	43 (100)
	**Total (%)**	**70 (39.5)**	**26 (14.7)**	**6 (3.4)**	**3 (1.7)**	**60 (33.9)**	**2 (1.1)**	**4 (2.3)**	**3 (1.7)**	**1 (0.6)**	**1 (0.6)**	**1 (0.6)**	**177 (100)**

Table S7. Continued, 80mm.

Diameter (mm)	Activity	Distal-proximal location											
		1	2	3	4	5	6	7	8	9	10	11	Total
**105**	**Static thumb adducted (%)**	34 (49.3)	4 (5.8)	4 (5.8)	1 (1.4)	20 (29.0)	1 (1.4)	3 (4.3)	2 (2.9)	0 (0)	0 (0)	0 (0)	69 (100)
	**Static thumb abducted (%)**	32 (49.2)	5 (7.7)	5 (7.7)	0 (0)	22 (33.8)	0 (0)	1 (1.5)	0 (0)	0 (0)	0 (0)	0 (0)	65 (100)
	**Dynamic (%)**	44 (72.1)	3 (4.9)	3 (4.9)	1 (1.6)	9 (14.8)	0 (0)	0 (0)	1 (1.6)	0 (0)	0 (0)	0 (0)	61 (100)
	**Total (%)**	**110 (56.4)**	**12 (6.2)**	**12 (6.2)**	**2 (1.0)**	**51 (26.2)**	**1 (0.5)**	**4 (2.1)**	**3 (1.5)**	**0 (0)**	**0 (0)**	**0 (0)**	**195 (100)**

Table S7. Continued, 105mm.

Table S8. Suspension radial-ulnar peak pressure location risk ratios calculated from the corresponding multinomial logistic regression. Reference categories are: 3rd Ray, Diameter 80, Activity Static thumb adducted.

	Dependent variable:		
	1st Ray	2nd Ray	4th Ray
	(1)	(2)	(3)
Diameter45	0.00000	0.311**	3.452***
	(418.979)	(0.466)	(0.332)
Diameter105	1.506	3.196***	0.00000
	(0.435)	(0.332)	(348.758)
ActivityDynamic	2.359**	1.813*	0.805
	(0.429)	(0.336)	(0.405)
ActivityStatic thumb abducted	0.00000	0.212***	1.182
	(633.965)	(0.406)	(0.351)
Constant	0.217***	0.302***	0.171***
	(0.359)	(0.293)	(0.338)
Akaike Inf. Crit.	731.258	731.258	731.258
Note:			*p **p ***p<0.01

Biology Open • Supplementary information

Table S9. Suspension distal-proximal peak pressure location risk ratios calculated from the corresponding multinomial logistic regression. Reference categories are: Proximal phalanges, Diameter 80, Activity Static thumb abducted.

	Dependent variable:			
	Distal phalanges	Intermediate phalanges	Metacarpal bases	Metacarpal heads
	(1)	(2)	(3)	(4)
Diameter45	0.077***	0.270***	0.000***	0.000
	(0.549)	(0.406)	(0.0001)	(669.261)
Diameter105	5.025***	0.468	2.807**	0.585
	(0.418)	(0.638)	(0.446)	(0.465)
ActivityDynamic	1.104	0.650	9.299***	6.658***
	(0.564)	(0.623)	(0.554)	(0.543)
ActivityStatic thumb adducted	0.906	1.099	0.676	0.522
	(0.388)	(0.401)	(0.496)	(0.461)
Constant	0.683	0.612	0.493*	1.199
	(0.352)	(0.367)	(0.392)	(0.334)
Akaike Inf. Crit.	865.557	865.557	865.557	865.557
Note:				*p **p ***p<0.01

Biology Open • Supplementary information

Table S10. Vertical climbing radial-ulnar peak pressure location risk ratios calculated from the corresponding multinomial logistic regression. Reference categories are: 3rd Ray, Diameter 105, Activity Static thumb adducted.

	Dependent variable:		
	1st Ray	2nd Ray	4th Ray
	(1)	(2)	(3)
Diameter45	0.00002	0.0002	2,959,805,837,692.000***
	(74.833)	(14.136)	(0.154)
Diameter80	0.826	1.909**	1,534,186,158,051.000***
	(0.490)	(0.274)	(0.170)
ActivityDynamic	79,337,069,063.000***	1.155	2.238**
	(0.235)	(0.326)	(0.345)
ActivityStatic thumb abducted	50,136,971,608.000***	0.987	2.354***
	(0.256)	(0.321)	(0.327)
Constant	0.000***	0.344***	0.000***
	(0.182)	(0.257)	(0.159)
Akaike Inf. Crit.	804.922	804.922	804.922
Note:			*p **p ***p<0.01

Biology Open • Supplementary information

Table S11. Vertical climbing distal-proximal peak pressure location risk ratios calculated from the corresponding multinomial logistic regression. Reference categories are, Distal phalanges, Diameter 105, Activity Static thumb adducted.

	Dependent variable:	
	Intermediate phalanges	Metacarpal bases
	(1)	(2)
Diameter45	36,410.140	3.867***
	(14.518)	(0.280)
Diameter80	8,264.076	1.821**
	(14.518)	(0.247)
ActivityDynamic	0.503*	0.675
	(0.368)	(0.268)
ActivityStatic thumb abducted	0.804	1.331
	(0.361)	(0.256)
Constant	0.0001	0.473***
	(14.518)	(0.225)
Akaike Inf. Crit.	833.021	833.021
Note:		*p **p ***p<0.01

Table S12. Suspension and vertical climbing sample sizes by diameter and activity for participant mean data points and usable raw data.

Diameter	Activity			
	**Thumb adducted static**	**Thumb abducted static**	**Mean static**	**Dynamic**
**Suspension**				
**45mm**				
Participant mean	14	14	14	13
Raw data	54	47	-	41
**80mm**				
Participant mean	14	14	14	14
Raw data	58	53	-	51
**105mm**				
Participant mean	12	13	12	13
Raw data	33	57	-	39
**Vertical Climbing**				
**45mm**				
Participant mean	14	14	14	14
Raw data	55	52	-	51
**80mm**				
Participant mean	14	14	14	14
Raw data	73	61	-	43
**105mm**				
Participant mean	14	13	13	14
Raw data	69	65	-	61

Biology Open • Supplementary information
